# Rain‐Induced Stratification of the Equatorial Indian Ocean and Its Potential Feedback to the Atmosphere

**DOI:** 10.1029/2021JC018025

**Published:** 2022-03-07

**Authors:** Kyle Shackelford, Charlotte A. DeMott, Peter Jan van Leeuwen, Elizabeth Thompson, Samson Hagos

**Affiliations:** ^1^ Department of Atmospheric Science Colorado State University Fort Collins CO USA; ^2^ Department of Meteorology University of Reading Reading UK; ^3^ NOAA Physical Sciences Lab Boulder CO USA; ^4^ Pacific Northwest National Laboratory Richland WA USA

**Keywords:** Surface freshening, air‐sea interaction, tropical convection, ocean stratification

## Abstract

Surface freshening through precipitation can act to stably stratify the upper ocean, forming a rain layer (RL). RLs inhibit subsurface vertical mixing, isolating deeper ocean layers from the atmosphere. This process has been studied using observations and idealized simulations. The present ocean modeling study builds upon this body of work by incorporating spatially resolved and realistic atmospheric forcing. Fine‐scale observations of the upper ocean collected during the Dynamics of the Madden‐Julian Oscillation field campaign are used to verify the General Ocean Turbulence Model (GOTM). Spatiotemporal characteristics of equatorial Indian Ocean RLs are then investigated by forcing a 2D array of GOTM columns with realistic and well‐resolved output from an existing regional atmospheric simulation. RL influence on the ocean‐atmosphere system is evaluated through analysis of RL‐induced modification to surface fluxes and sea surface temperature (SST). This analysis demonstrates that RLs cool the ocean surface on time scales longer than the associated precipitation event. A second simulation with identical atmospheric forcing to that in the first, but with rainfall set to zero, is performed to investigate the role of rain temperature and salinity stratification in maintaining cold SST anomalies within RLs. Approximately one third, or 0.1°C, of the SST reduction within RLs can be attributed to rain effects, while the remainder is attributed to changes in atmospheric temperature and humidity. The prolonged RL‐induced SST anomalies enhance SST gradients that have been shown to favor the initiation of atmospheric convection. These findings encourage continued research of RL feedbacks to the atmosphere.

## Key Points

- A 1D water column model reproduces observed temperature and salinity stratification and rain layer (RL) statistics for the equatorial Indian Ocean
- Experiments with and without rain falling onto the ocean show that RLs sustain cold sea surface temperature (SST) anomalies by resisting vertical mixing
- RLs persist beyond formative rain events, enhancing spatial SST gradients that can generate atmospheric boundary layer convergence

## 1 Introduction

Upper ocean stratification is the result of processes that form or advect low‐density water layers on top of higher‐density water layers. Stratification is affected by many processes, including upwelling, wind stirring, warming from solar radiation, salinification due to evaporation, and surface freshening from precipitation and river run‐off (Asher et al., 2014; Bellenger & Duvel, 2009; Drushka et al., 2016; Hughes et al., 2020; Kraus & Turner, 1967; Soloviev & Lukas, 1997; Thompson et al., 2019). Ocean stratification regulates vertical mixing of heat, nutrients, and gases, and affects climatologically important low‐frequency ocean processes, such as the formation of North Atlantic deep water (Broecker, 1991), carbon uptake (Watson et al., 2020), and the El Niño‐southern Oscillation (Cronin & McPhadden, 2002).

On shorter time scales, near‐surface stable layers induced by the diurnal cycle of surface solar heating are classified as diurnal warm layers (DWLs) while those induced by freshwater fluxes from rainfall are classified as rain layers (RLs), or freshwater lenses. Stabilization within RLs and DWLs shoals (i.e., makes more shallow) the ocean mixed layer and reduces vertical mixing between the near‐surface ocean and the ocean mixed layer by altering upper ocean temperature and salinity profiles. Through their ability to resist vertical mixing, these shallow stable layers may then confine subsequent surface inputs of heat, momentum, and freshwater to the upper 1–10 m of the ocean. Changes to sea surface temperature (SST) and sea surface salinity (SSS) within these near‐surface stable layers modify fluxes of heat, moisture, and momentum across the air‐sea interface.

The spatially broad and temporally regular nature of DWLs has allowed for extensive study of these phenomena, and their impact upon atmospheric convection is well‐documented (Bellenger & Duvel, 2009; Bellenger et al., 2010; de Szoeke et al., 2021). Increased SST within DWLs deepens the atmospheric boundary layer and helps regulate the diurnal cycle of convection in the tropics, and inclusion of DWL parameterizations in atmospheric models has improved forecasting of the MJO (Woolnough et al., 2007; Zhao & Nasuno, 2020) and ENSO (Masson et al., 2012; Terray et al., 2012), indicating that DWLs contribute to climate variability on the intraseasonal and interannual time scales. However, while DWLs are the result of diurnal surface heating that is often quasi‐uniform over large scales, RLs are the result of intermittent precipitation which can be highly irregular for a given location. As such, less is known about the spatiotemporal characteristics of RLs or their cumulative effects on the atmosphere.

While multiple studies record changes to upper ocean profiles within DWLs (Bellenger & Duvel, 2009; Fairall, Bradley, Godfrey, et al., 1996; Hughes et al., 2020; Soloviev & Lukas, 1997; Stuart‐Menteth et al., 2003), knowledge of how RLs adjust upper ocean salinity and temperature profiles, as well as air‐sea exchange, has been limited by observational constraints. Currently, operational satellites tasked with measuring SSS include the Soil Moisture, Active/Passive (SMAP; Vinogradova et al., 2019), with a 40 km footprint and 2–3 days revisit time, and the Soil Moisture Ocean Salinity (SMOS; Vinogradova et al., 2019), with a 43 km footprint and 3–5 days revisit time, which are too infrequent and spatially coarse to capture the impacts of convective scale to mesoscale surface freshening (DeMott & Rutledge, 1998; Richenback & Rutledge, 1998). Moorings provide nearly continuous observations at the coarse horizontal resolution, but their coarse vertical resolution of the upper ocean prevents investigation of the near‐surface impacts of RLs. Similarly, operational Argo floats are usually limited by coarse upper ocean vertical resolution, as well as coarse horizontal and temporal sampling (Gould et al., 2004). The most useful observations for investigating RLs have been provided by field campaigns, which allow for ship‐based, collocated ocean‐atmosphere observations, with frequent sampling and fine‐scale vertical resolution. However, field campaigns are held infrequently and for limited duration, thus limiting the direct observation of changes to SSS, SST, and surface fluxes within RLs.

Thompson et al. (2019) used upper ocean observations collected in the equatorial Indian Ocean as part of the Dynamics of the Madden‐Julian Oscillation field campaign (DYNAMO; Yoneyama et al., 2013) to study near‐surface stabilization in DWLs and RLs. They found that while the freshening and cooling of the upper ocean have opposing effects on stability, the positive buoyancy produced by freshening is generally about an order of magnitude greater than the negative buoyancy produced by cooling. Additionally, they observed that RL‐induced buoyancy is strong enough to withstand nocturnal ocean convective mixing and wind‐driven mixing for wind speeds up to 9.8 m s^−1^ for the heaviest rain rates. Mean RL lifetime observed by Thompson et al. (2019) was 5 hr, with some RLs lasting nearly a full day. Thus, the typical RL lifetime is longer than the typical lifetime of rain events that initiate RLs (Hagos et al., 2013).

RL persistence on the scale of hours suggests that RL lifetimes are long enough to impact the atmospheric boundary layer (DeMott et al., 2015; de Szoeke et al., 2017), but RL feedback to atmospheric convection is not straight‐forward. Locally, RLs stabilize and cool the upper ocean, potentially hindering the initiation of new convection (Ruppert & Johnson, 2016) and reducing the maintenance of existing convection by surface fluxes (Riley Dellaripa & Maloney, 2015). However, sharp SST gradients exist between the RL and surrounding ocean, generating horizontal pressure gradients that act to initiate boundary layer convergence and stimulate atmospheric convection (Back & Bretherton, 2009a, 2009b; Li & Carbone, 2012; Rydbeck et al., 2019; Skyllingstad et al., 2019). The nature of the atmospheric response to RL formation remains an important open question for understanding the impact of freshwater ocean surface stratification on atmospheric convection.

Recently, idealized model experiments have increased understanding of RL characteristics, revealing the importance of rain rate, wind speed, and background ocean stratification in regulating RL behavior (Drushka et al., 2016; Iyer & Drushka, 2021b; Soloviev et al., 2015). While experiments investigating RLs in an idealized environment have provided insight into upper ocean response to precipitation, the collective effects of RLs under realistic, time‐varying atmospheric forcing on SST patterns, surface fluxes, and feedbacks to atmospheric convection is less understood. We aim to address this knowledge gap with a modeling study designed to answer the following science questions:

1. What is the size, frequency, duration, and intensity of equatorial Indian Ocean RLs on monthly time scales?
2. To what extent do RLs alter surface fluxes and create small‐scale networks of SST gradients?

To address these questions, a 1‐dimensional water column model is used to simulate freshwater stratification in the equatorial Indian Ocean. The design of the model simulations is discussed in Section 2. The model is first verified when forced with surface observations collected during DYNAMO and compared to observed ocean stability profiles. Results from this analysis are shown in Section 3. After model verification, a 50 × 50 km 2D array of 1D columns is forced with surface meteorology at 2 km resolution from an existing simulation of the regional atmospheric Weather Research and Forecasting model (WRF; Skamarock et al., 2019). Stratification by surface freshening is analyzed to determine spatial and temporal characteristics of RLs that result from the multitude of spatially and temporally inhomogeneous, model‐simulated rain and wind events. We present results from this analysis in Sections 4 and 5, and further discuss the implications of these results in Section 6. In Section 7, we conclude with a brief summary that highlights the primary conclusions of this study.

## 2 Methods

In this section, the General Ocean Turbulence Model (GOTM) is introduced (Section 2.1). The specifics of two model simulations are discussed, where the first simulation serves the purpose of model verification (Section 2.2), while the second is used to generate statistics describing RL characteristics and variability of upper ocean stability (Section 2.3).

### 2.1 Model Configuration

GOTM is a water‐column model that computes solutions for the one‐dimensional version of the transport equations of momentum, salt, and heat (Burchard et al., 1999). The version of GOTM implemented in this study closely follows the model setup of Drushka et al. (2016), which has been shown to effectively replicate upper ocean temperature and salinity response to rainfall. This version of GOTM utilizes a second‐order turbulence closure scheme (Canuto et al., 2001) with dynamic dissipation rate equations for the length scales. Fluxes are calculated following the Coupled Ocean‐Atmosphere Response Experiment bulk flux algorithm (Fairall, Bradley, Rogers, Edson, & Young, 1996), which uses skin temperature to compute surface fluxes. Longwave radiation is calculated following Clark et al. (1974). GOTM assumes wet bulb temperature for rainfall, which is supported by observations (Gosnell et al., 1995). The model is run with a 10 s time step and initialized to a depth of 70 m with 10 cm vertical resolution. GOTM's sensitivity to upper ocean vertical resolution was tested at vertical resolutions of 1, 10, 50, and 100 cm, and negligible improvement was seen in model performance at vertical resolution below 10 cm. GOTM receives surface forcing input in the form of horizontal components of the 10 m winds, and surface values of air temperature, air pressure, relative humidity, incident shortwave radiation, and rain rate. In this study, GOTM is forced first using observations collected during the DYNAMO field campaign (Gottschalck et al., 2013; Yoneyama et al., 2013), and then using WRF model output from a 2014 study by Hagos et al. (2014). The details of the surface forcing data are outlined in the following sections.

### 2.2 Model Verification and DYNAMO Data

The DYNAMO field campaign was conducted in the Indian Ocean from October 2011 through March 2012, with the purpose of observing convective initiation processes associated with the MJO. The field campaign was an international effort featuring two quadrilateral sounding arrays, multiple radars, simultaneous and continuous observations of atmospheric and oceanic profiles conducted from three moorings and two ships (research vessel (R/V) Roger Revelle and R/V Mirai), twin sites in the Indian Ocean (Addu Atoll) and Western Pacific (Manus Island) to sample the MJO at its initiation, mature, and dissipating phases, and an aircraft operation for sampling atmospheric and oceanic coupled boundary layers (Chen et al., 2016; Yoneyama et al., 2013).

Of interest for this study are the high‐frequency atmospheric and oceanic observations collected from the R/V Revelle (80.5°E, 0°N) during the 5 October through 30 October and 11 November through 7 December 2011 DYNAMO observing periods. Each period sampled one full MJO event (Gottschalck et al., 2013), and thus featured a broad spectrum of ocean‐atmospheric variability, from strongly suppressed and light‐rain conditions to highly disturbed and heavy‐rain conditions. The observations from the R/V Revelle are unique in that they feature collocated ocean‐atmosphere observations that are high‐resolution both temporally (upper ocean profiles observed at roughly 7 min intervals), and vertically (1 m resolution for upper ocean observations; Moum et al., 2014). The fine vertical resolution of the upper ocean observations, which begin at a depth of 2–3 m (Thompson et al., 2019), allows for detailed comparisons of GOTM output to observations made from the R/V Revelle. Additionally, the frequent nature of the observations allows for transient rain events to be effectively captured within both meteorological surface data and near‐surface ocean temperature and salinity profiles. Thompson et al. (2019) analyzed these same observations to study RLs and DWLs.

**Figure 1:**
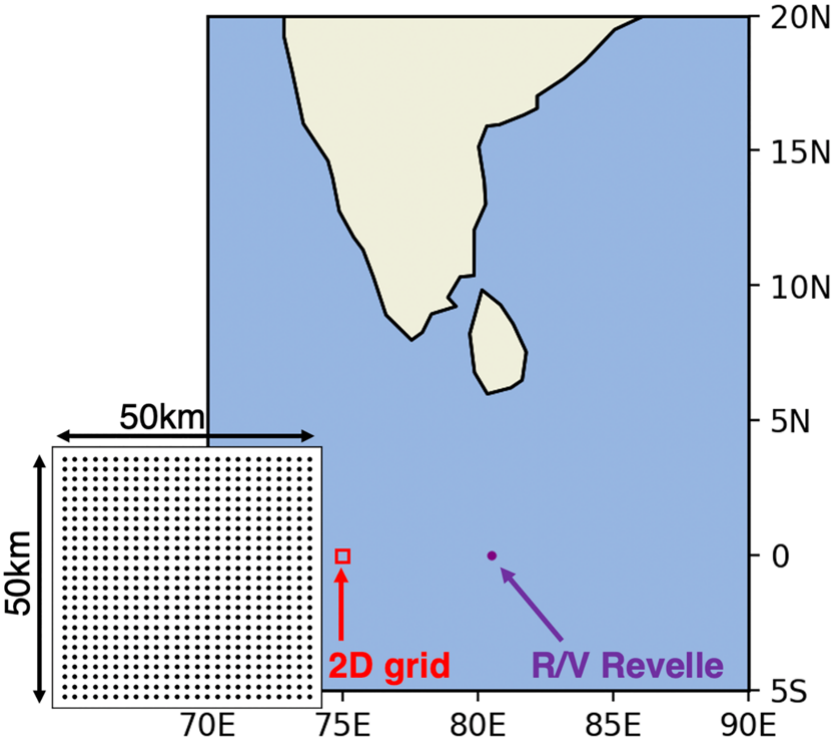
Model domain for the two General Ocean Turbulence Model simulations: the location of the R/V Revelle during Dynamics of the Madden‐Julian Oscillation field campaign (purple) and domain for the 2D array experiment (red box). The inset grid displays the dimensions and layout of the 2D array.

For the first DYNAMO simulation, GOTM is initialized with temperature and salinity profiles from the R/V Revelle on 6 October 2011 at 01:30:00 UTC, and is then forced with atmospheric observations at 10 min intervals until 12:00:00 UTC on 30 October (Figure 1). For the second DYNAMO simulation, GOTM is initialized with temperature and salinity profiles from the R/V Revelle on 11 November 2011 19:20:00 UTC and is again forced with atmospheric observations at 10 min intervals until 8 December 2011 05:30:00 UTC. No relaxation to an observed or climatological mean temperature and salinity profile was needed to replicate the observed upper ocean conditions. Each simulation captures observed intraseasonal variability attributable to the MJO, with conditions varying from fair weather with low cloudiness, light rainfall, calm winds, and high incident shortwave radiation during the MJO suppressed phase to deep and widespread cloudiness, heavy precipitation, strong winds, and reduced surface solar radiation during the MJO disturbed phase, enabling comparison of GOTM model output to observations under diverse conditions.

For each DYNAMO simulation, GOTM stability profiles are calculated using vertical gradients in potential density to find the Brünt‐Vaisala frequency, *N* ^2^:

(1) $$N^{2}=\frac{g}{\sigma }\frac{d\sigma }{dz}$$

As salinity and temperature both play important roles in ocean stratification, upper ocean stability as indicated by *N* ^2^ is governed by vertical gradients of both quantities. To view temperature (*T*) and salinity (*S*) contributions to *N* ^2^ separately, *N* ^2^ can be decomposed into its temperature and salinity components, $N_{T}^{2}$ and $N_{S}^{2}$, defined as:

(2) $$N_{T}^{2}=g\cdot \alpha \cdot \frac{dT}{dz}$$

(3) $$N_{S}^{2}=g\cdot \beta \cdot \frac{dS}{dz}$$

where *β* is the haline contraction coefficient of seawater, and *α* is the thermal expansion coefficient of seawater:

(4) $$\beta =\frac{1}{\sigma }\frac{d\sigma }{dS}$$

(5) $$\alpha =\frac{-1}{\sigma }\frac{d\sigma }{dT}$$

This decomposition allows for identification of RLs, which are present when the upper ocean is stably stratified with respect to both total *N* ^2^ and it's salinity component, $N_{S}^{2}$, and DWLs, which are present when the upper ocean is stably stratified with respect to both total *N* ^2^ and it's temperature component, $N_{T}^{2}$. Following the methods of Thompson et al. (2019), the column is considered to be stable if *N* ^2^ > 4.5 × 10^−5^ s^−1^ for two consecutive vertical layers; otherwise, the column is considered well‐mixed. Modeled *N* ^2^ values are computed every 0.5 m for the upper 20 m of the ocean; thus, minimum thickness for a layer to be considered stable is 1 m. For both observational and model analysis, the upper ocean is considered well‐mixed if no stable layers are identified in the upper 20 m of the ocean. Observations synthesized by Thompson et al. (2019) were used to validate model output. Since observations are collected with a vertical resolution of 1 m, the column is considered stable for two consecutive 1 m layers and the minimum thickness of observed stable layers is 2 m, compared to a minimum thickness of 1 m for modeled stable layers.

### 2.3 WRF Data and GOTM 2D Array

For the second portion of this study, a 2D array of GOTM columns is forced with model output from a WRF simulation conducted by Hagos et al. (2014). WRF was run at 2 km horizontal resolution over a 3° × 3° latitude‐longitude area within the Indian Ocean DYNAMO domain from 1 October 2011 to 30 November 2011. This grid spacing is fine enough to resolve individual convective systems while the domain is large enough and the simulation long enough to capture lifecycles of convective systems associated with synoptic scale features (Chen et al., 1996; Hagos et al., 2014) This makes the WRF data well‐suited for this study: the 2 km grid spacing resolves atmospheric convective‐scale forcing of the upper ocean, while the spatial domain allows for the development of organized mesoscale convective systems that contribute to the variability of surface forcing. Surface boundary conditions in the WRF simulation are provided by ERA‐Interim reanalysis, and applied at 6 hr intervals. Comparison between WRF precipitation output and TRMM satellite observations shows that while WRF is able to capture the overall eastward propagation of the two MJO events during October and November 2011, the model precipitation tends to be higher than TRMM observations. Hagos et al. (2014) attribute this discrepancy to the model resolution, which limits turbulent mixing and evaporation of rain. Further details on the WRF parameterizations can be found in Hagos et al. (2014) (Figure 1).

To investigate spatiotemporal variability of RLs, the 2D array of GOTM columns is forced with output from the WRF simulation over a 50 × 50 km grid, centered over 75°E, 0°N with 2 km grid spacing. This 50 × 50 km domain allows investigation of fine‐scale spatial variability of upper ocean temperature and salinity profiles, SST, and SSS on scales smaller than those currently resolved by most global models and satellite‐estimated SSS products. Initial conditions for the temperature and salinity profile at each grid cell are obtained from the Hybrid Coordinate Ocean Model (HYCOM) reanalysis data set (Chassignet et al., 2007). HYCOM provides vertical temperature and salinity at depths of 0.05, 2, 4, 6, 8, 10, 12, 15, 20, 25, 30, 35, 40, 45, 50, 60, 70 m, which are then linearly interpolated by GOTM to a 10 cm grid spacing from 0 to 70 m. The more coarse horizontal grid spacing in the HYCOM reanalysis (0.08°) is linearly interpolated to the WRF grid. Analysis of the DYNAMO simulations shows GOTM sensitivity to small variations in initial temperature and salinity profiles to be small compared to variations introduced via surface forcing. The 2D array simulation is run from 00 UTC 1 November 2011 to 18 UTC 30 November 2011. GOTM is again forced at 10 min intervals with no relaxation to a reference temperature or salinity profile.

We use GOTM output from the 2D array simulation to conduct a statistical analysis detailing RL characteristics. The 2D domain allows for analysis of RL characteristics under spatially variable wind and rain forcing over a typical MJO lifecycle, for a satellite footprint‐sized domain. To conduct this analysis, we first use the stable layer identification algorithm described in Section 2.2 to detect RLs. We then investigate RL behavior as a function of rain rate, wind speed, and background ocean stratification (Section 4.1). In Section 4.2, we make approximations of RL size as determined by RL equivalent diameter and in Section 4.3, we analyze reduced mixing in RLs using the temperature tendency equation in GOTM. Finally, we examine the potential for RLs to influence the atmospheric boundary layer by repeating the second simulation over the 2D domain without rain forcing (i.e., rain rate, *R* = 0 everywhere, for all time steps) but with all other atmospheric forcing fields identical (Section 5).

## 3 Model Verification: Comparisons to DYNAMO Observations

While previous studies have verified the ability of GOTM to simulate observed upper ocean response to precipitation (Drushka et al., 2016), upper ocean observations from the R/V Revelle allow for a more detailed comparison of stable layers analyzed by Thompson et al. (2019) to those simulated by GOTM. Furthermore, the October and November DYNAMO legs provide an opportunity to evaluate GOTM performance under different background conditions, as an advection event brought high salinity water from the Arabian Sea into the DYNAMO domain between the October and November observing periods. The high‐salinity water mass contributed to the formation of a barrier layer that was present throughout the November observation period.

**Figure 2:**
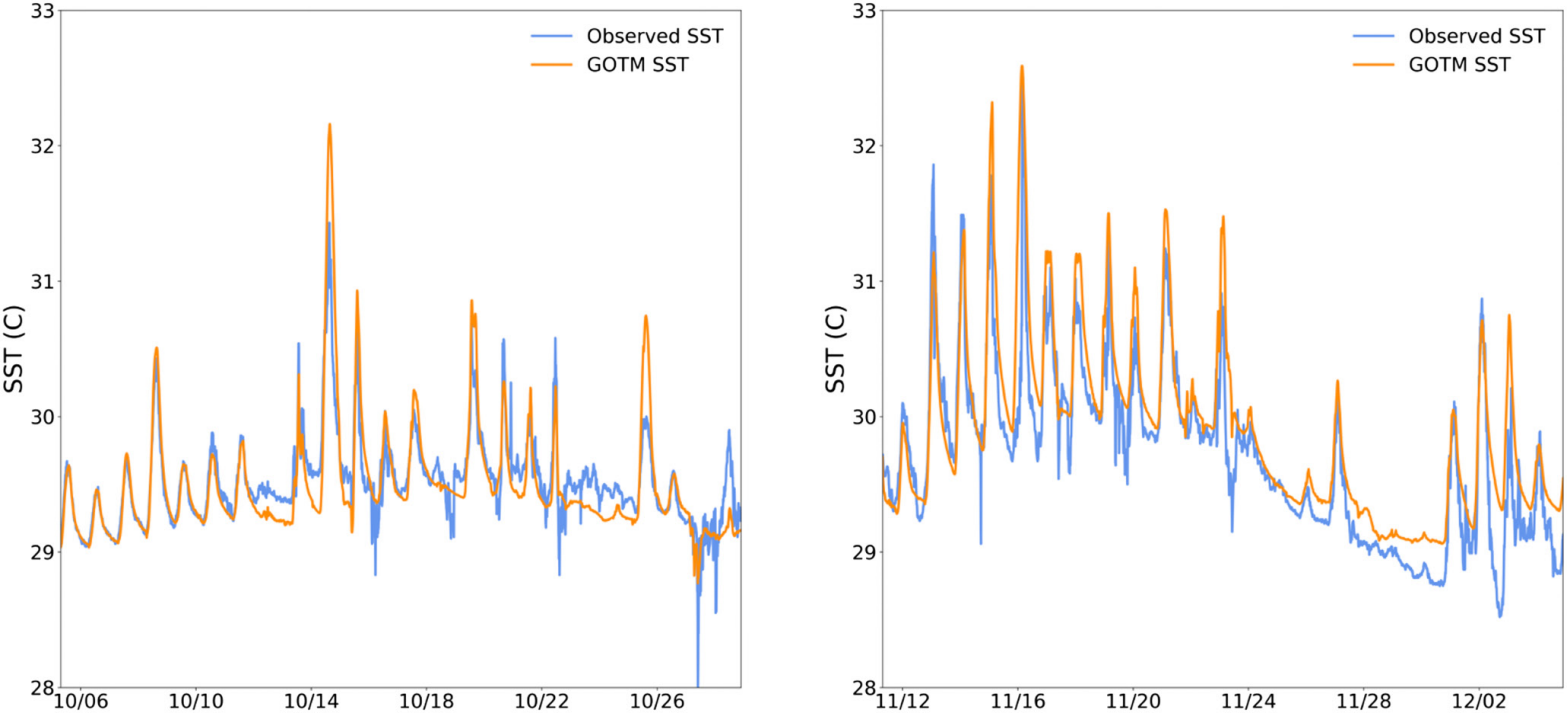
Modeled (orange) and observed (blue) sea surface temperature (SST) time series for the October (left) and November (right) Dynamics of the Madden‐Julian Oscillation field campaign observing periods.

Initial steps in model verification involve comparison of GOTM temperature and salinity profiles to profiles observed during the October and November DYNAMO legs. Time series comparison between GOTM SST and observed SST for both simulations confirms that the model effectively replicates the strong diurnal cycle of SST during suppressed MJO conditions, as well as the reduced, diurnally uniform SST during active MJO conditions and westerly wind burst (WWB) events (Figure 2). Modeled SST mean absolute error for the October and November observing periods is 0.14°C and 0.24°C, respectively.

**Figure 3:**
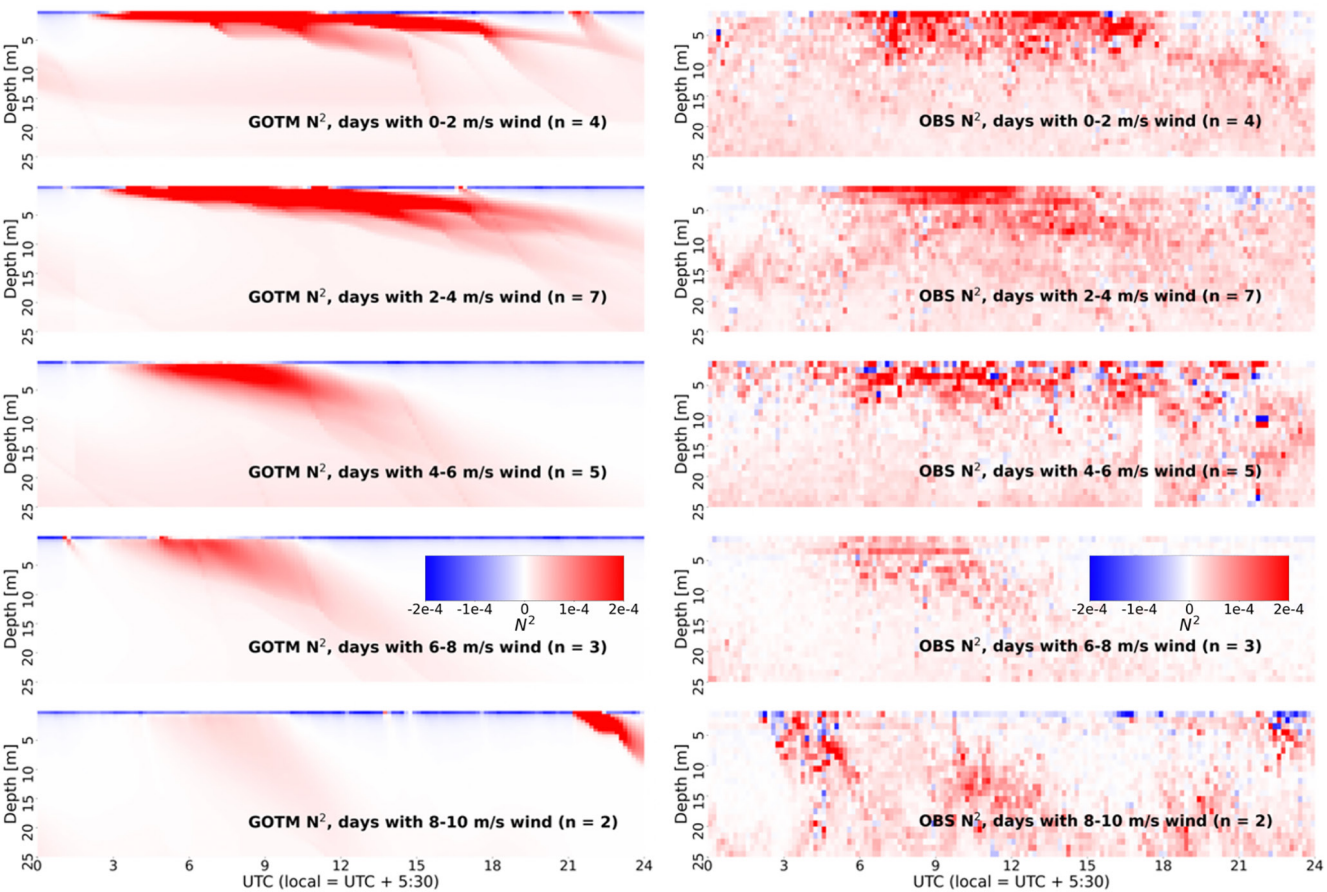
Daily $N_{T+S}^{2}$ profiles composited by daily wind regime for General Ocean Turbulence Model GOTM, left and observations (right). Red indicates stable, blue indicates unstable. Number of days within a given wind regime is given by *n*.

Composite analysis is used to make qualitative comparisons between *N* ^2^ profiles computed using model output and observations for the October and November DYNAMO observing periods. For the composite analysis, each 24 hr, 1‐day period in the simulation is binned by daytime mean wind speed in intervals of 2 m s^−1^, and the mean *N* ^2^, $N_{T}^{2}$, and $N_{S}^{2}$ profiles are computed for each wind regime. These composites are then compared to observed *N* ^2^, $N_{T}^{2}$, and $N_{S}^{2}$ profiles, that is, those computed by Thompson et al. (2019) from DYNAMO temperature and salinity observations. Each wind speed interval tends to include multiple days, thus, the composite *N* ^2^ profiles are dominated by DWLs and the effects of individual precipitation events are generally not detected. However, this enables a more general evaluation of model performance in capturing changes in vertical stratification as a function of wind speed. Composite *N* ^2^ profiles shown in Figure 3 demonstrate that GOTM mixing reproduces stratification characteristics of different wind regimes: a persistent, shallow, and strong diurnal signature is present on days with calm winds, a diminished, but deeper, diurnal signature is present on days with moderate winds, and virtually no diurnal signal is detectable on the windiest days, when turbulent mixing is too strong for ocean stratification to develop. Model performance is consistent across the October and November simulations, thus verifying the ability of GOTM to effectively reproduce upper ocean stability profiles under different background ocean stratification. Profiles of *N* ^2^ variability computed from GOTM also agree well with those observed during DYNAMO (not shown).

**Figure 4:**
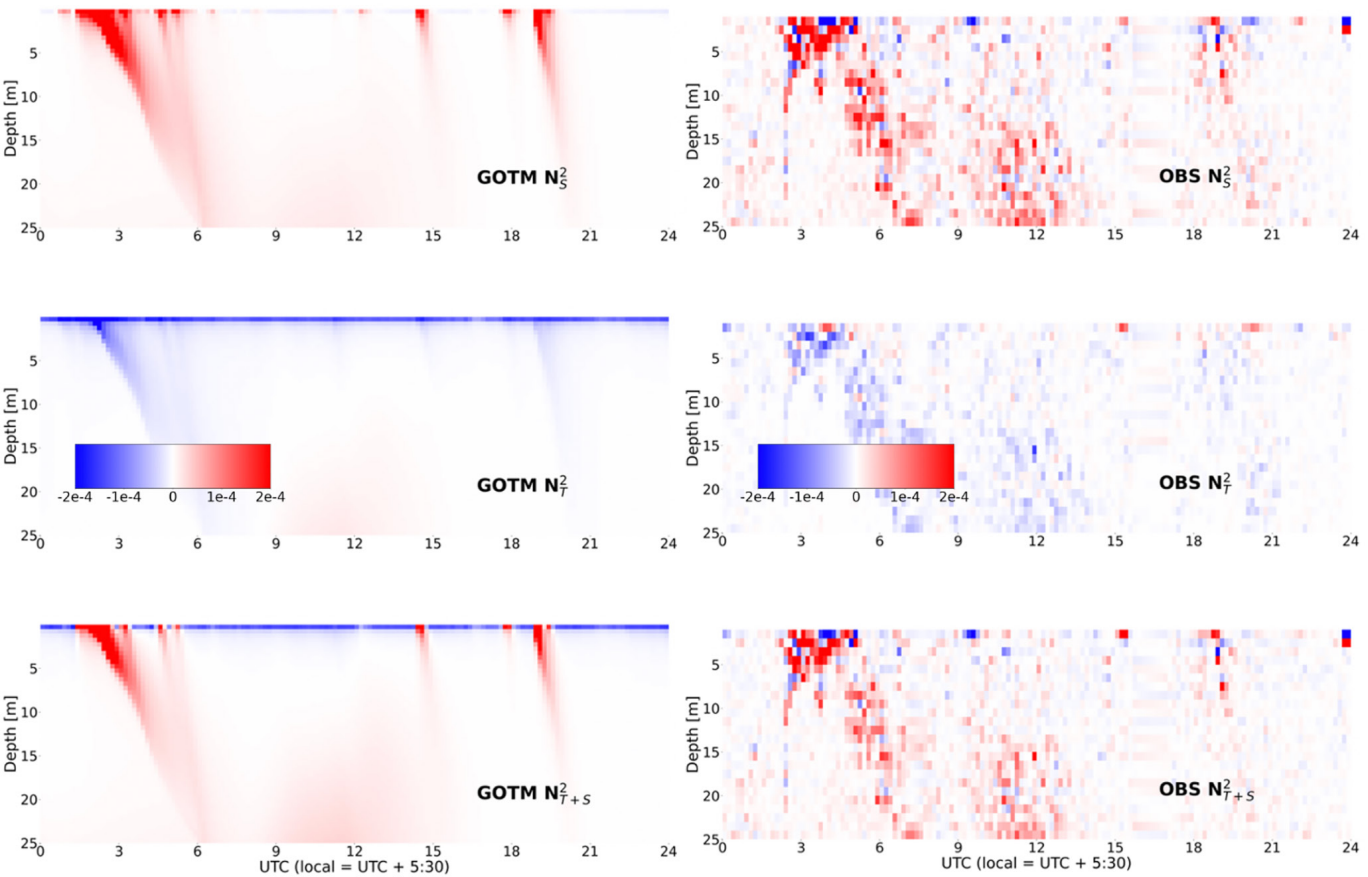
$N_{S}^{2}$ (top), $N_{T}^{2}$ (middle), $N_{T+S}^{2}$ (bottom), for 28 November 2011 for General Ocean Turbulence Model (GOTM, left) and observations (right). Red indicates stable, blue indicates unstable.

In order to assess model performance in simulation of individual freshening events, an RL detection algorithm is utilized to identify RLs, following criteria outlined in Section 2.2 of this article. It is important to note that salinity observations recorded during DYNAMO begin at a depth of 2–3 m, due to interference from the ship's wake in the upper 2 m of the ocean. Thus, it is useful to evaluate freshening events associated with high precipitation amounts and >2 m s^−1^ wind speed, as these freshening events generally affect the $N_{S}^{2}$ profile to a depth greater than 2 m. One such case of an RL event with a strong signature below 2 m is found in DYNAMO observations from 28 November when an RL was identified from 02 UTC to 05 UTC. Observed and modeled stability profiles for the 28 November case can be seen in Figure 4. The 28 November RL developed following sustained precipitation of >10 mm hr^−1^, and during a period of reduced wind speeds (<10 m s^−1^) within a longer WWB event, allowing a short‐lived RL to form. Figure 4 demonstrates that GOTM is able to reproduce the onset, stabilization, and duration of the observed RL. 28 November is of further interest as a daily case study due to the sustained precipitation that occurred throughout the day. The high winds present during the day prevented sustained stratification of the upper ocean, but Figure 4 reveals multiple brief stratification events in GOTM stability profiles. These highly transient RLs coincide with temporary reductions in wind speed seen throughout the day that allowed for the upper 1–2 m of the ocean to become stably stratified. While some of these stratification events are evident in observations, the lack of salinity observations in the upper 2 m inhibits identification of the thinnest RLs.

## 4 RL Statistics From 2D Forcing Experiments

This section applies results from the 2D array simulation to produce statistics defining spatiotemporal characteristics of equatorial Indian Ocean RLs. The array of GOTM columns is forced at 10 min intervals with output from a WRF simulation conducted by Hagos et al. (2014); see Section 2.3. We emphasize that these are not ocean‐atmosphere coupled simulations. The output from the WRF simulation is simply used to force the 2D GOTM array, and any changes to SST are not communicated to the atmosphere.

For the 2D array simulation, the RL identification algorithm iterates grid cell by grid cell searching the upper 3 m of the ocean for RLs at each time step. Because each GOTM column mixes independently from neighboring columns, we consider each RL‐capped column as a separate, distinct RL when computing RL duration, frequency, and intensity statistics (Section 4.1). However, we consider adjacent RL‐capped columns to be part of a single, larger RL when computing RL size statistics (Section 4.2).

### 4.1 RL Duration, Frequency, and Intensity

**Figure 5:**
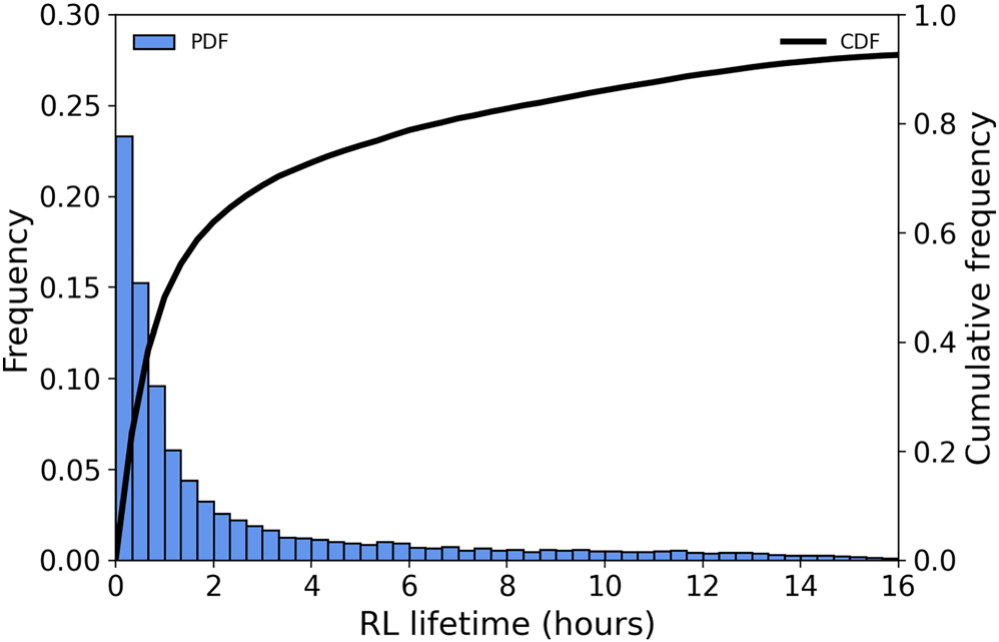
Histogram of rain layer (RL) lifetime frequency, binned by 20 min intervals (left *y*‐axis), and cumulative frequency (black line; right *y*‐axis).

The duration of RLs over the course of the simulation is highly variable, with modeled RLs persisting on time scales of minutes to days. The distribution of RL lifetime is skewed, as 32% of RLs last less than 30 min, 48% last less than 1 hr, and 96% last less than 1 day (Figure 5). Mean RL duration is roughly 4.5 hr, which conforms to statistics of RL lifetimes observed during DYNAMO (Thompson et al., 2019), while median RL duration is just over 1 hr. Although RLs occur at all times, there is a slight increase in RL formation in the early morning and mid‐afternoon, which is consistent with the mean diurnal cycle of precipitation over tropical oceans during convectively active and suppressed conditions, respectively (Sui et al., 1997). Overall, RLs are present in 26% of all model time steps, a higher frequency than the 16% observed by Thompson et al. (2019) during DYNAMO.

**Figure 6:**
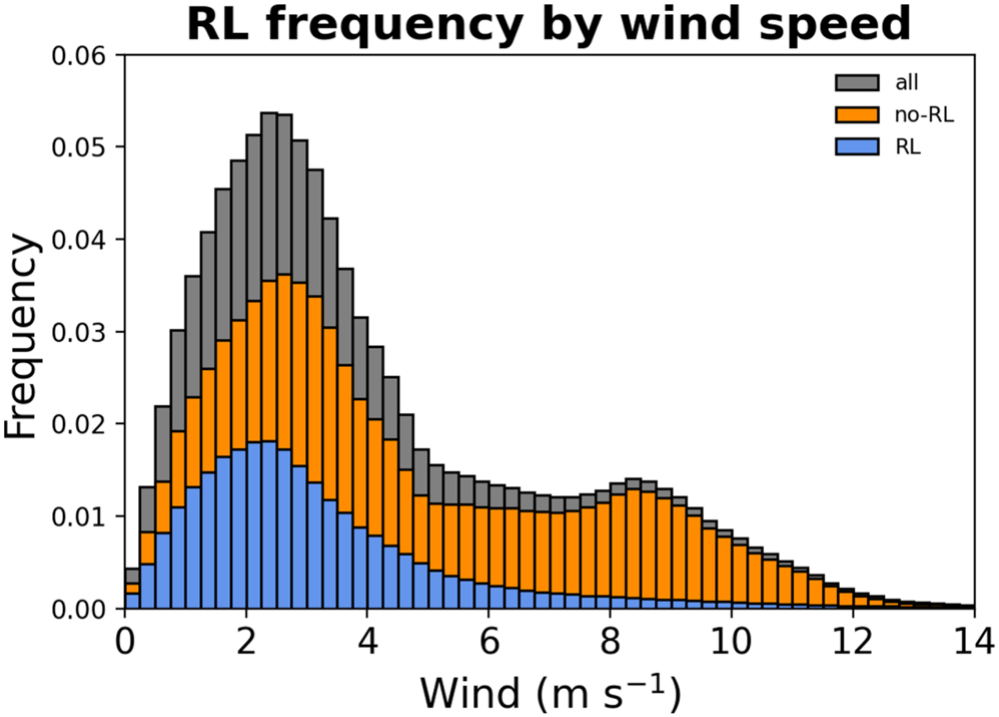
Histogram of wind speed frequency across all General Ocean Turbulence Model (GOTM) grid cells and times when a rain layer (RL) is present (blue), no‐RL is present (orange), and overall (gray).

During periods of low to moderate winds, RLs persist for several hours to over a day, and occur more frequently (Figure 6). This is reflected by an RL presence of 32% for time steps when column wind speed is <5 m s^−1^, in comparison to an RL presence of 14% for time steps when column wind speed is >5 m s^−1^. The 99th percentile wind speed in the presence of RLs is 11.4 m s^−1^, slightly greater than the 99th percentile wind speed of 9.8 m s^−1^ observed by Thompson et al. However, the 95th percentile wind speed in the presence of RLs is 7.97 m s^−1^, indicating that rain‐induced stratification at wind speeds above 8 m s^−1^ is typically short‐lived. When no RLs are present, the 95th percentile wind speed is 10.1 m s^−1^. The large discrepancy between 95th percentile wind speeds in the presence (7.97 m s^−1^) and absence (10.1 m s^−1^) of RLs implies that RLs occur infrequently at wind speeds above 8 m s^−1^.

**Figure 7:**
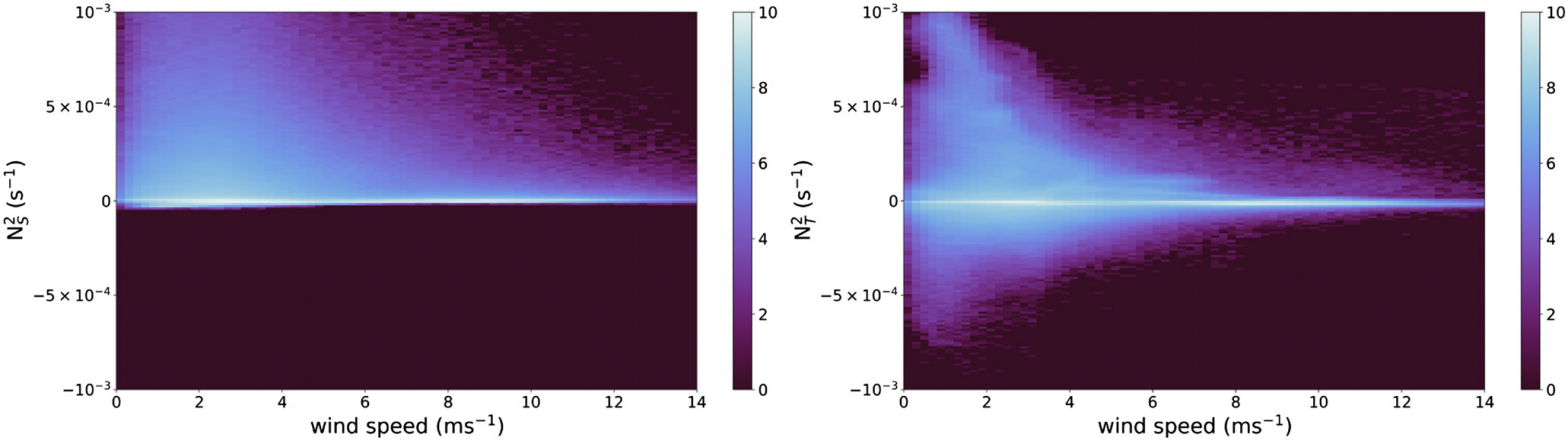
2D histogram of $N_{S}^{2}$ and wind (left) and $N_{T}^{2}$ and wind (left). Histograms display the natural log value of the count within each bin.

Stability profiles of temperature and salinity in the upper ocean are sensitive to wind speed, with the strongest stabilization of both occurring most frequently at wind speeds below 5 m s^−1^. Histograms of $N_{S}^{2}$ and $N_{T}^{2}$ as a function of wind speed for all model time steps and grid cells are shown in Figure 7, and reveal a higher frequency of strong stability in the salinity profile in comparison to the temperature profile. The higher frequency of strong stabilization in the salinity profile is especially evident at higher wind speeds, which is consistent with observational analysis of RLs and DWLs (Thompson et al., 2019).

**Figure 8:**
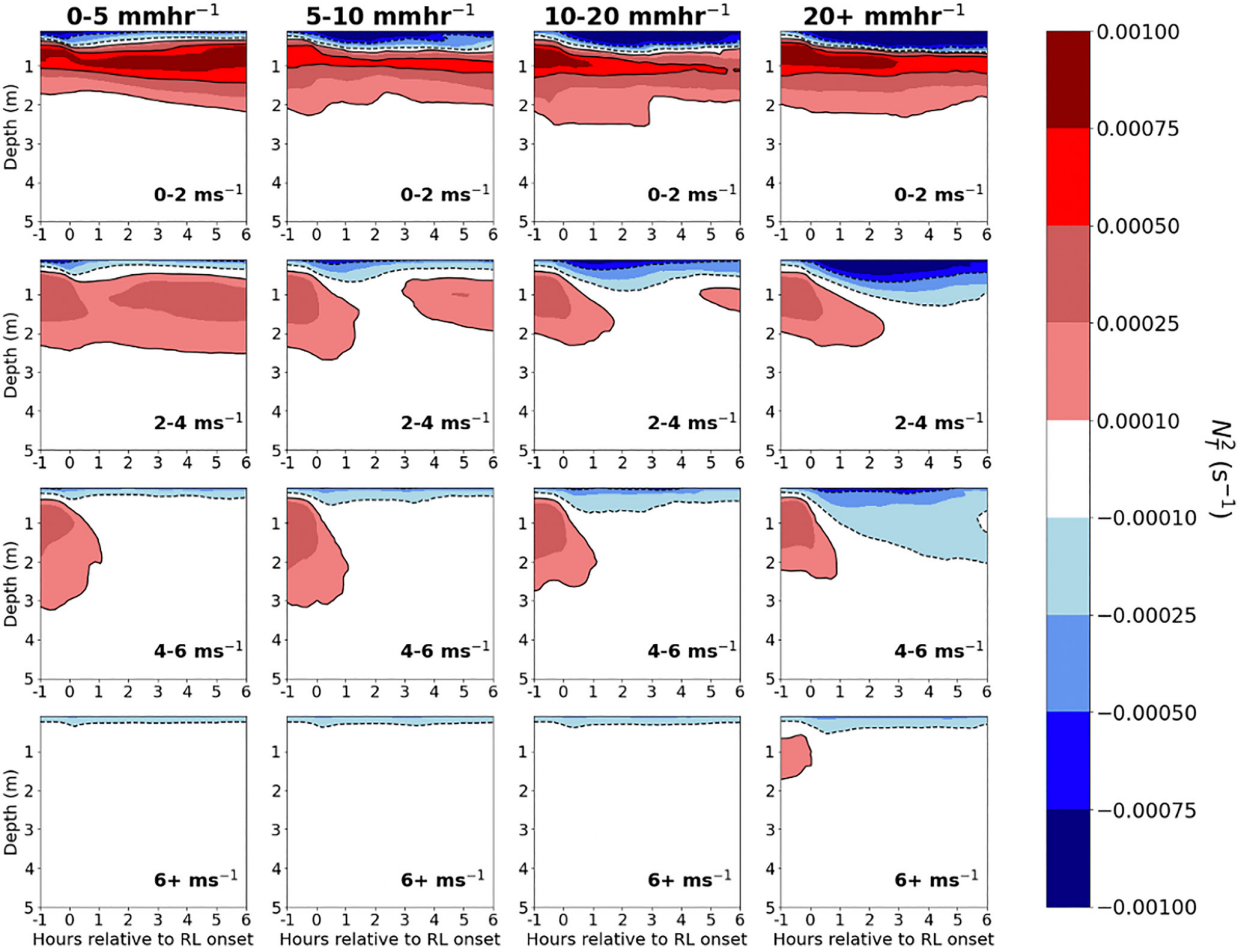
$N_{T}^{2}$ within rain layers (RLs) binned column‐wise by max rain rate from 30 min prior to 30 min after RL formation, and row‐wise by mean wind speed over the interval of 1 hr prior to 6 hr after RL formation. Note the unstable *T* profiles at the ocean surface within the RLs.

Instability can be identified within the temperature profile at wind speeds below 5 m s^−1^ Figure 7, a result of both nocturnal convective mixing and unstable temperature profiles within RLs. Composite analysis of the $N_{T}^{2}$ response from one hour prior to RL formation to six hours after RL formation as a function of the mean wind speed and maximum rain rate in the ±1 hr interval surrounding RL onset is presented in Figure 8, and confirms destabilization in column temperature profiles following RL formation. For RLs forming under background wind speeds <6 m s^−1^, unstable temperature gradients confined to the upper 1–2 m persist for many hours following RL formation (Figure 8). The persistence of unstable temperature gradients is due to a stronger stabilization of $N_{S}^{2}$, which is also reflected in a net positive *N* ^2^ throughout the column (not shown).

**Figure 9:**
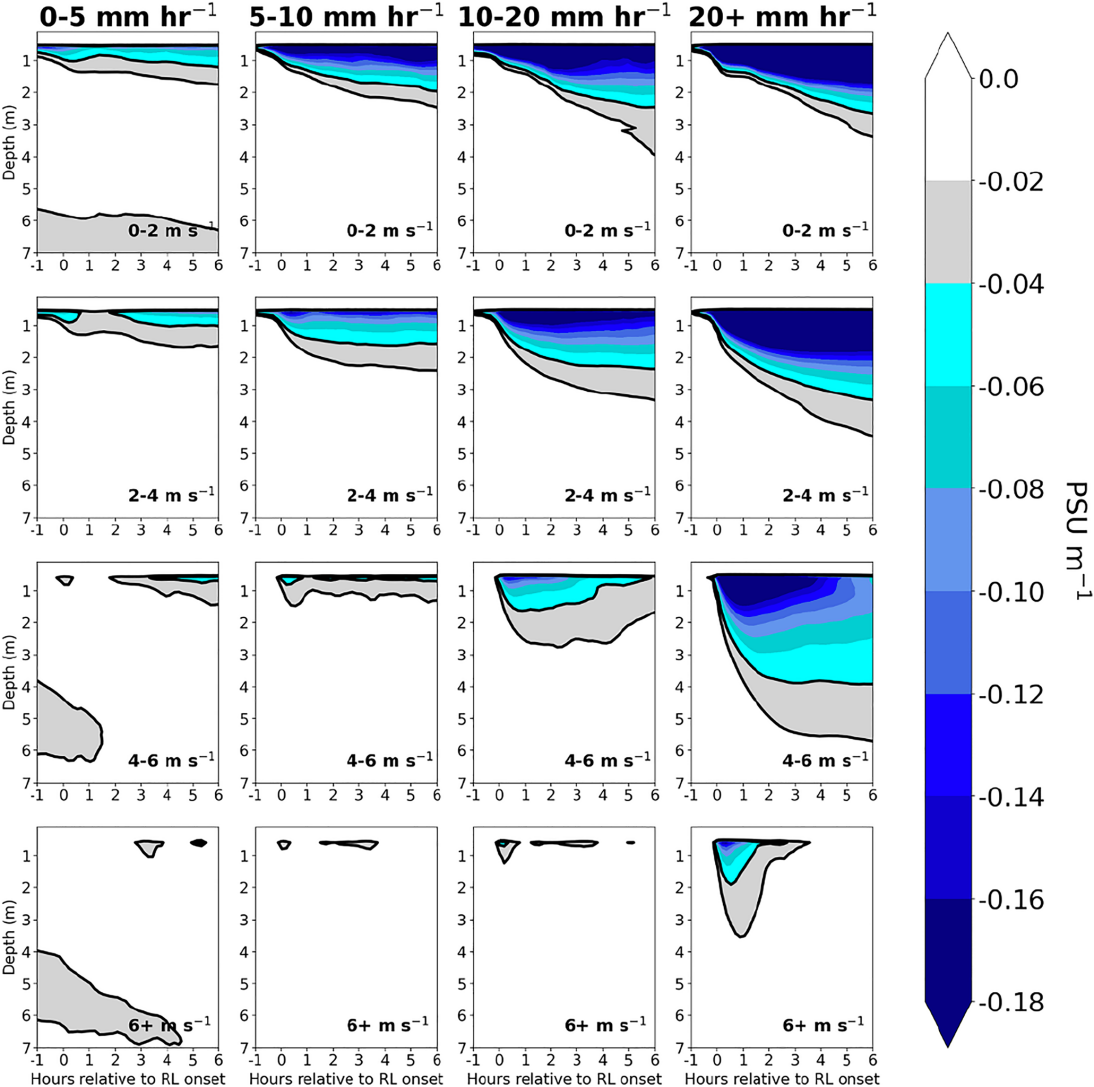
Salinity gradient $\left(\frac{\partial S}{\partial z}\right)$ within rain layers (RLs) binned column‐wise by max rain rate from 60 min prior to 60 min after RL formation, and row‐wise by mean wind speed over the interval of 1 hr prior to 1 hr after RL formation. Salinity gradient is computed as the centered difference (PSU m^−1^) at 1 m intervals, and thus begins at a depth of 0.5 m.

Because the vertical salinity gradient and $N_{S}^{2}$ within RLs are constrained by surface inputs of freshwater and momentum, the magnitude of stabilization in RLs is primarily determined by rain rate and wind speed. Figure 9 displays the composite evolution of the salinity gradient from one hour prior to RL formation to six hours after RL formation. For a given wind speed (i.e., panels in a single row in Figure 9), the magnitude and depth of the upper ocean salinity gradient increases with increasing rain rate, reflecting a higher degree of stabilization within RLs forming under stronger rain rates. The impact of wind speed on RL formation is also evident in columns of fixed rain rate, as the magnitude of the upper ocean salinity gradient within RLs decreases with increasing wind speed. At wind speeds above 6 m s^−1^, typically only the strongest rain rate cases are able to stratify the upper ocean for more than an hour, consistent with previous observations and theory (Thompson et al., 2019).

It is noteworthy that RLs forming under weak rain rates (<5 mm hr^−1^) and weak surface winds (0–2 and 2–4 m s^−1^), feature a persistent, stable vertical salinity gradient confined to the upper 1–2 m of the ocean (Figure 9). While the magnitude of stabilization is reduced in weak rain rate cases compared to stronger rain rate cases, the stable salinity gradient in these cases is able to persist for many hours following RL formation. The implications of long‐lasting RLs under low surface wind conditions are revisited in Section 5.

**Figure 10:**
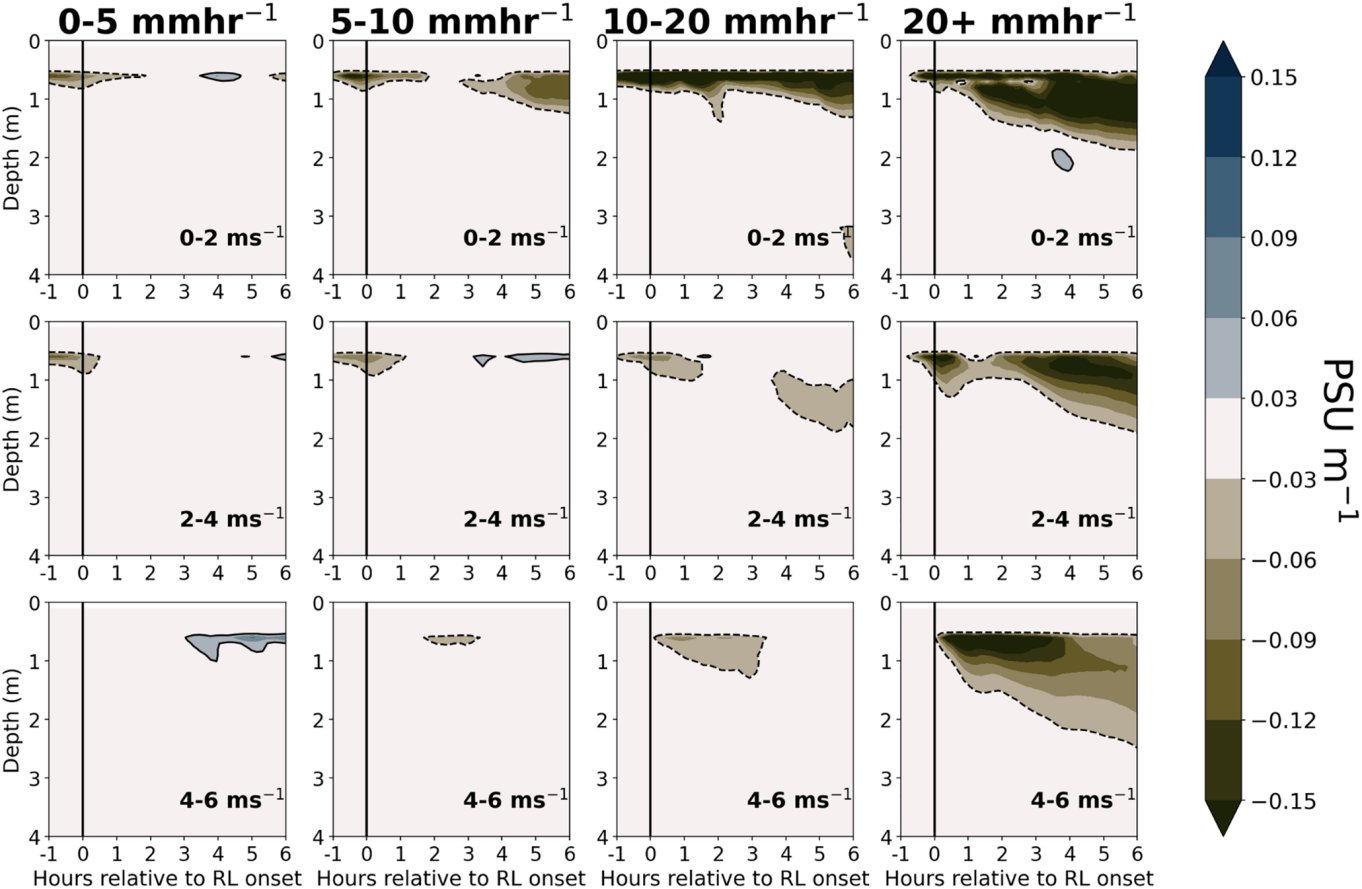
Difference in salinity gradient between rain layers (RLs) forming over a strongly stratified upper ocean with respect to ($N_{T}^{2}>$ 1 × 10^−4^) and all RLs, from one hour prior to six hours after RL formation. Brown shading (negative) represents a stronger salinity gradient in RLs forming over a strongly stratified upper ocean, while blue shading (positive) represents a weaker salinity gradient in RLs forming over a strongly stratified upper ocean. Figure is binned column‐wise by max rain rate from 60 min prior to 60 min after RL formation, and row‐wise by mean wind speed over the interval of 1 hr prior to 1 hr after RL formation.

When precipitation falls on a stably stratified upper ocean, vertical mixing of freshwater is further inhibited, resulting in RLs that feature a strong vertical salinity gradient and that are even more persistent than RLs that form over a well‐mixed upper ocean. Figure 10 shows the composite difference in salinity gradient between RLs forming over a strongly stratified upper ocean with respect to $N_{T}^{2}$, defined as mean $N_{T}^{2}\;>$ 1 × 10^−4^ s^−2^ in the upper 5 m of the column, compared to the salinity gradient for all other RLs. Within the same rain rate and wind speed bins, RLs forming over an upper ocean that is strongly stratified with respect to $N_{T}^{2}$ feature a more intense salinity gradient in comparison to all other RLs (Figure 10). As there are few cases of RLs forming over strong upper ocean stratification at wind speeds above 6 m s^−1^, wind speed bins in Figure 10 only extend to 6 m s^−1^. This result conforms to the idealized model experiments of Iyer and Drushka (2021a) that revealed larger salinity anomalies and delayed mixing in the upper ocean when rain falls on a stably stratified upper ocean compared to rain falling on a well‐mixed upper ocean.

### 4.2 RL Spatial Dimensions

**Figure 11:**
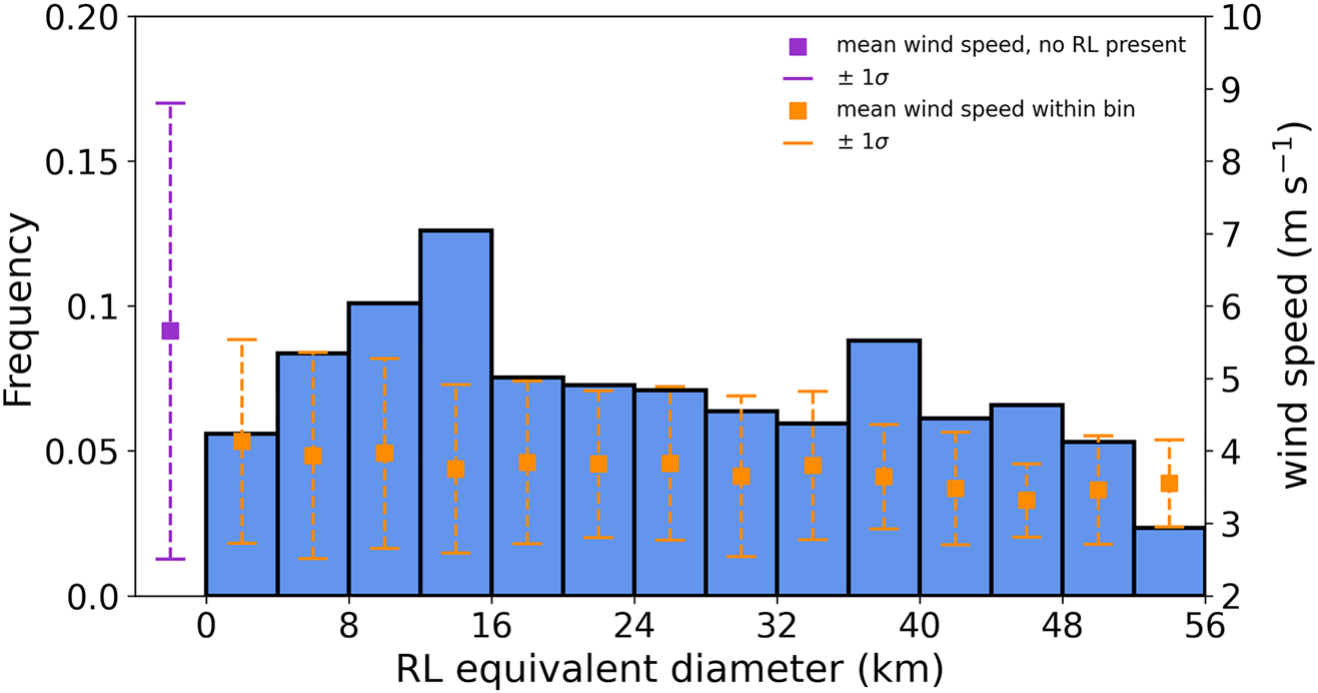
Histogram of RL equivalent diameter frequency (blue), with domain‐averaged wind speed ± 1*σ* overlaid for the corresponding bin (orange). RL equivalent diameter represents equivalent diameter of largest contiguous RL for time steps when RLs are present. The *x*‐axis has been extended to include the domain‐averaged wind speed ± 1*σ* for time steps when no RLs are present (purple).

The footprint of contiguous cells with RLs ranges from as small as a single 2 × 2 km grid cell to as large as 97% of the 50 × 50 km domain. For purposes of estimating RL footprint size, the maximum number of adjacent grid points containing an RL for a given time step is computed, and a distribution of RL equivalent diameter is determined. Figure 11 shows the frequency of RL equivalent diameter, with possible values of RL equivalent diameter spanning 2–55.6 km. For time steps in which RLs are present, the mean and median RL equivalent diameter of the largest RL present are 25 and 23 km, respectively.

Evaluating domain‐averaged wind speed within each RL equivalent diameter bin in Figure 11 shows consistent values of mean domain‐averaged wind speed across all RL sizes. Within RL equivalent diameter bins, mean values of domain‐averaged wind speed range from 3.32 m s^−1^ (RL equivalent diameter 44–48 km) to 4.13 m s^−1^ (RL equivalent diameter 0–4 km), compared to a mean domain‐averaged wind speed of 5.65 m s^−1^ when no RLs are present. However, wind speed variability within each bin decreases with increasing equivalent diameter, indicating that the largest RL footprints are less likely to occur at higher wind speeds.

### 4.3 Reduced Vertical Mixing Within RLs

In order to quantify the degree of mixing within RLs, the temperature tendency term in GOTM is decomposed into contributions from solar radiation and contributions from the sum of turbulent and viscous transport. The temperature tendency term in GOTM for a given level is defined as:

(6) $$\dot{\theta }=\frac{\partial }{\partial z}\left(\left(\nu_{T}^{\theta }+\nu^{\theta }\right)\frac{\partial \theta }{\partial z}\right)+\frac{1}{C_{p}\rho_{0}}\frac{\partial I}{\partial z}$$

where $\dot{\theta }$ is the material derivative of potential temperature, $\nu_{T}^{\theta }$ and *ν* ^*θ*^ are the turbulent and molecular diffusivities of heat, respectively, *C*_*p*_ is the heat capacity of seawater, and *ρ*_0_ is a reference density (Burchard et al., 1999). Shortwave radiation, *I* is prescribed and treated as an inner heat source as a function of depth, *z*. The source due to shortwave radiation is computed by GOTM according to a double exponential law following Paulson and Simpson (1977), assuming Jerlov type I water. The sum of latent heat, sensible heat, and longwave radiation fluxes are computed by GOTM at each time step and is treated as a boundary condition for *∂θ*/*∂z*. Thus, the first term on the right hand side of Equation 6 represents temperature tendency from turbulent and viscous transport, and the second term represents a source term from shortwave radiation. We compute the profile of temperature tendency from transport as the difference between the total temperature tendency profile and the shortwave heating profile.

**Figure 12:**
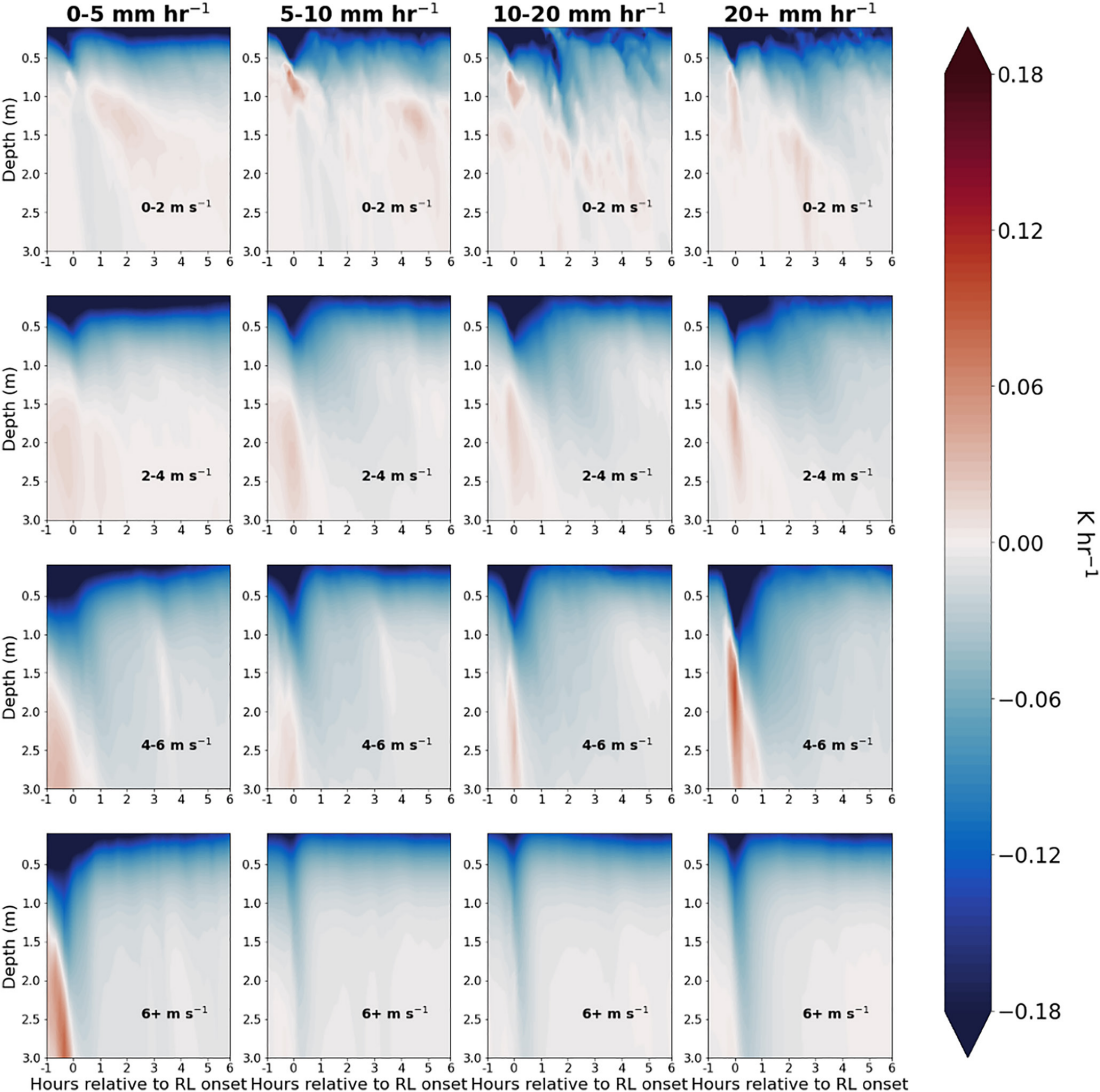
Temperature tendency due to transport from one hour prior to six hours after rain layer (RL) formation. Figure is binned column‐wise by max rain rate from 60 min prior to 60 min after RL formation, and row‐wise by mean wind speed over the interval of 1 hr prior to 1 hr after RL formation.

The vertical profile of temperature tendency due to transport over the course of RL lifetime as a function of wind speed at rainfall rate is shown in Figure 12. The negative tendency due to transport in the upper 1–2 m of the column immediately preceding and following RL formation is associated with decreased air temperature and surface input of cool freshwater surrounding RL onset. Transport cooling persists from +1 to +6 hr following RL formation but over limited depth compared to the short RL onset period, despite an unstable temperature stratification in the 0.5–1 m (Figure 8). We revisit the reduction in transport mixing following RL onset in the following section.

## 5 The Potential for RL Feedbacks to the Atmosphere

Analyses shown in the previous section document the effects of RLs on ocean stability profiles. Here, we investigate the second science question posed in Section 1, namely, how RLs may affect the atmosphere. Ocean processes are communicated to the atmosphere through their effects on fluxes of heat, moisture, and momentum at the air‐sea interface. Since our 1D ocean model configuration assumes zero lateral advection, ocean processes in our experiments only regulate fluxes of heat and moisture by modulating the SST.

### 5.1 RL Regulation of SST and Surface Fluxes

**Figure 13:**
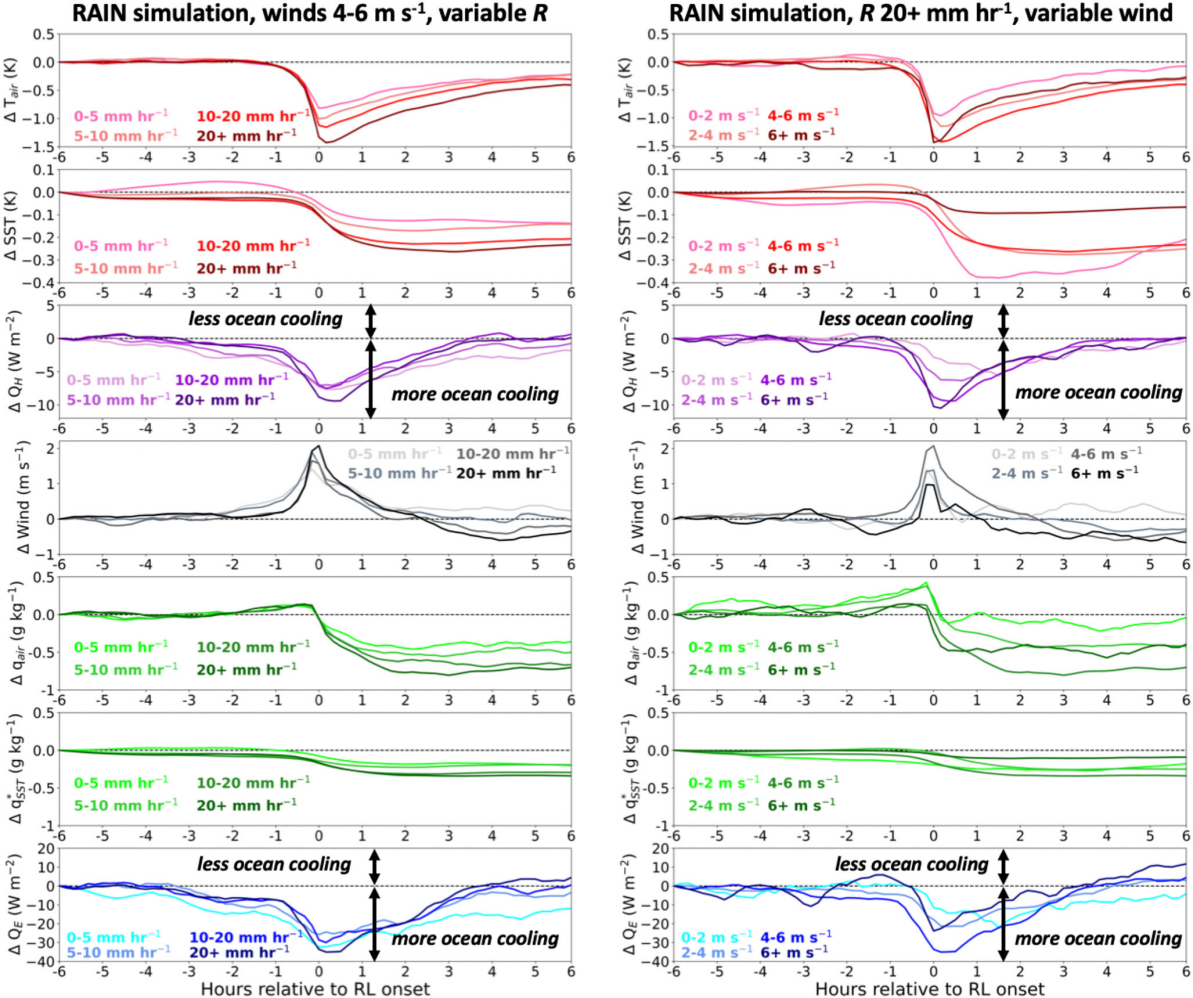
Mean departure from 6 hr preceding rain layer (RL) onset (hour −6) of (top to bottom): air temperature at 2 m (*T*_air_); SST; sensible heat flux (*Q*_*H*_); wind; specific humidity at 2 m (*q*_air_); saturation specific humidity at SST ($q_{air}^{*}$); and latent heat flux (*Q*_*E*_). In the left column, the mean wind speed surrounding RL onset is fixed between 4 and 6 m s^−1^, while rain rate varies; in the right column, the max rain rate preceding RL onset is fixed at >20 mm hr^−1^, while wind speed varies. Darkening color tone reflects increasing rain rate (left) and wind speed (right), for given bin. Fluxes are computed following Fairall, Bradley, Godfrey, et al., 1996 and Fairall, Bradley, Rogers, et al., 1996, and a negative Δ*Q*_*E*_ or Δ*Q*_*H*_ indicates greater ocean surface cooling.

Figure 13 displays the composite evolution of SST, air temperature at 2 m (*T*_air_), specific humidity at 2 m (*q*_air_), wind speed at 10 m, latent heat flux (*Q*_*E*_), and sensible heat flux (*Q*_*H*_) from six hours prior to six hours after RL formation. The sign convention for surface fluxes is that a negative flux or flux anomaly cools the ocean. First, we composite the aforementioned variables for RLs that form when the wind speed averaged from −1 hr prior to +1 hr following RL formation is 4–6 m s^−1^ (left column of Figure 13). Second, we composite the variables for RLs that form when the maximum *R* from −1 hr prior to +1 hr following RL formation exceeds 20 mm hr^−1^ (right column of Figure 13).

While both SST and *T*_air_ decrease following RL genesis, the decrease in *T*_air_ is nearly an order of magnitude larger than the decrease in SST. Consequently, the sensible heat flux becomes more negative following RL formation, reflecting a greater flux of sensible heat into the atmosphere from the ocean. Similarly, negative departures in the latent heat flux occur immediately preceding and following RL formation, and generally persist for 3–4 hr following RL formation. Consequently, for all RLs, the enhancements (i.e., more negative departures) in *Q*_*E*_ and *Q*_*H*_ surrounding RL onset are attributed to both the brief increase in wind speed at RL onset, as well as the more prolonged reductions in *T*_air_ and *q*_air_ following RL onset.

For RLs forming under a fixed background wind speed and different rain rates (left column of Figure 13), there is a systematic relationship between increasing rain rate, *R*, and larger negative departures of SST, *T*_air_, *q*_air_, *Q*_*E*_, and *Q*_*H*_ in the ±1 hr span surrounding RL onset. However, for the higher *R* events (*R* > 10−20 mm hr^−1^), *Q*_*H*_ is restored to pre‐RL values more rapidly than in low *R* events, and *Q*_*E*_ departures become positive beyond 4 hr following RL onset. The heat flux response is the result of a decreased wind speed following RL formation in high *R* events, as well as a larger reduction in SST in high rain rate cases than low *R* cases. A similar response is seen in RLs forming under a fixed maximum *R* and different wind speeds (right column of Figure 13), with increasing wind speed generally associated with enhanced *Q*_*E*_ and *Q*_*H*_ in the ±1 hr span surrounding RL onset. The overall relationship between wind speed and fluxes within RLs (right column of Figure 13) is more difficult to assess than the relationship between *R* and fluxes within RLs (left column of Figure 13), as there is large variability within the wind speed bins in the hours following RL onset.

To quantify the role of rainfall in regulating stratification, surface fluxes, and SST perturbations, a second GOTM simulation was conducted over the same domain using identical ocean surface forcing as the first, except all precipitation fluxes were set to zero. Hereafter, we refer to the simulations with and without rain forcing as “RAIN” and “NO‐RAIN”, respectively. Thus, while rain does not fall onto the ocean in the NO‐RAIN experiment, other forcing from the WRF output that is used in the RAIN experiment—*T*_air_, wind speed, *q*_air_, and net downwelling radiation—remains the same. Thus, any differences in stratification, surface fluxes, and SST between the two experiments arise purely from the presence of rainfall.

**Figure 14:**
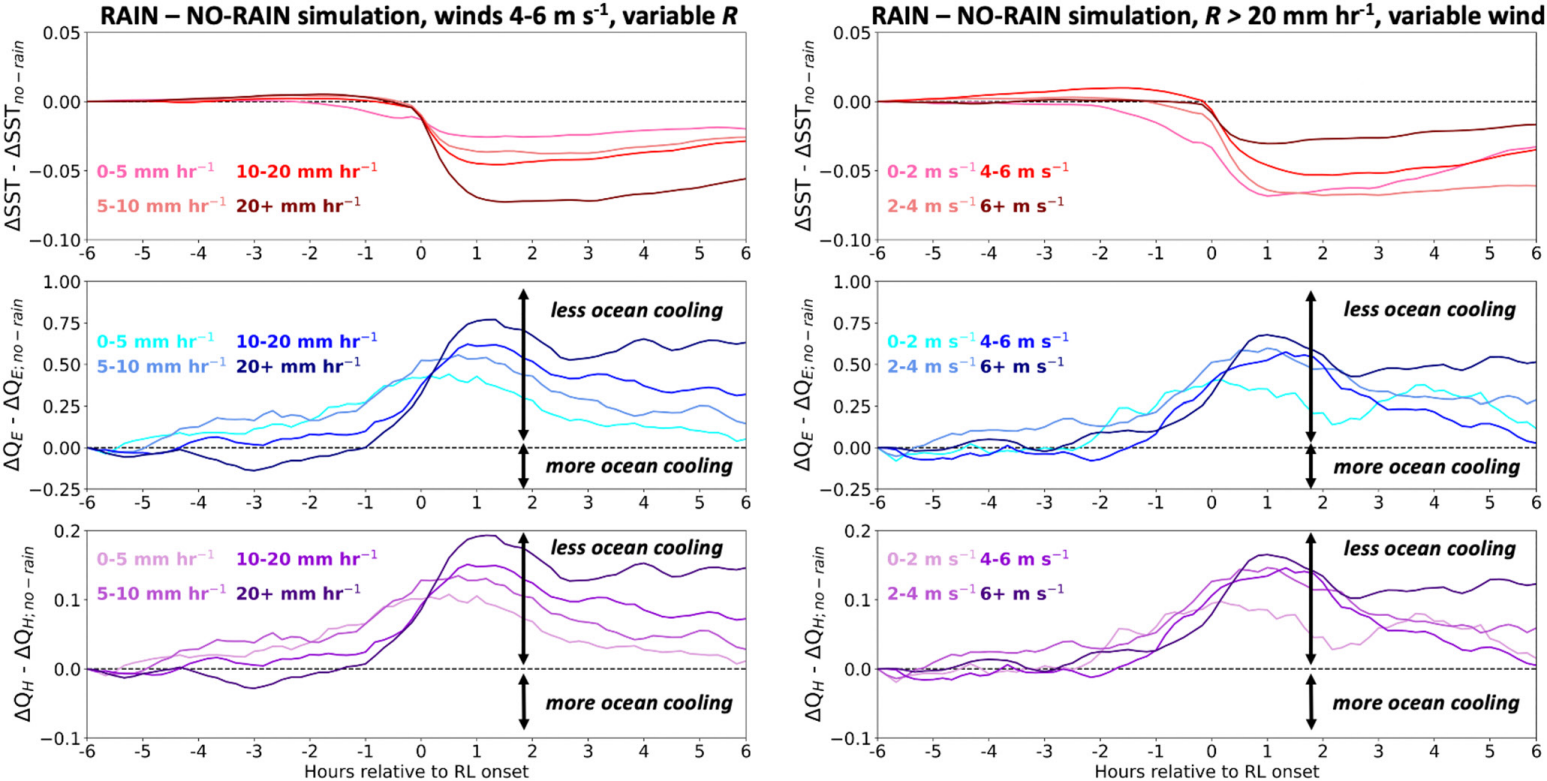
Difference between simulation with and without precipitation forcing from −6 hr to +6 hr relative to rain layer (RL) onset of sea surface temperature (SST) response from −6 hr (top, Kelvin), *Q*_*E*_ response from −6 hr (middle, Wm^−2^), and *Q*_*H*_ response from −6 hr (bottom, Wm^−2^). We note the change in vertical scale in all the plots. Note the persistent reduced SSTs following RL onset in the RAIN simulation in comparison to the NO‐RAIN simulation.

Figure 14 shows the difference in ΔSST, Δ*Q*_*E*_, and Δ*Q*_*H*_ from Figure 13 between the RAIN and NO‐RAIN experiments for all RLs binned by *R* (left column) and wind speed (right column). Across all *R* and wind speed bins, SSTs reduction for several hours following RL formation is 0.05–0.1 K greater in the RAIN experiment, and hence within actual RLs, relative to the NO‐RAIN experiment. Comparing the magnitude of SST reduction in RAIN (Figure 13) to that in NO‐RAIN (Figure 14), the combined effects of cooling and stratification by rainfall can be seen to account for approximately 30%–50% of the total SST reduction following RL onset. The influence of rain cooling on SST can be isolated to a rough approximated using wet‐bulb temperature, SST, rain amount, and RL thickness:

(7) $$\Delta T_{RL}\approx \left(T_{wetbulb}-SST\right)\cdot \frac{\text{rain amount}}{\text{RL thickness}}$$

Applying typical values of *T*_wetbulb_ − SST = −5 K (Thompson et al., 2019), rain amount = 10 mm, and RL thickness = 1 m to this equation, rain cooling alone can be estimated to reduce SST by 0.05 K.

The colder SST following RL onset in RAIN is reflected in the positive Δ*Q*_*E*_ and Δ*Q*_*H*_ differences in Figure 14, indicating weaker ocean‐to‐atmosphere surface fluxes compared to NO‐RAIN. Unlike SST, however, Δ*Q*_*E*_ and Δ*Q*_*H*_ between the two simulations differ by less than 2% following RL onset. This weak sensitivity of surface fluxes to RL‐induced SST changes is a consequence of the much larger reductions of *T*_air_ and *q*_air_ than SST and $q_{SST}^{*}$, respectively, following RL onset (Figure 13). The composite time evolution of *T*_air_ surrounding RL onset follows a pattern typical of atmospheric cold pools (de Szoeke et al., 2017; Figure 13).

The reduced SST following RL onset in RAIN occurs despite the slightly weaker post‐RL surface fluxes compared to those in NO‐RAIN. We surmise that the colder post‐RL SST in RAIN is the result of reduced downward transport of surface waters that have been cooled by the net heat transport out of the ocean. In essence, the salinity stratified RL in RAIN traps surface cooling within the RL, whereas cooled surface waters in NO‐RAIN are readily mixed throughout the column.

**Figure 15:**
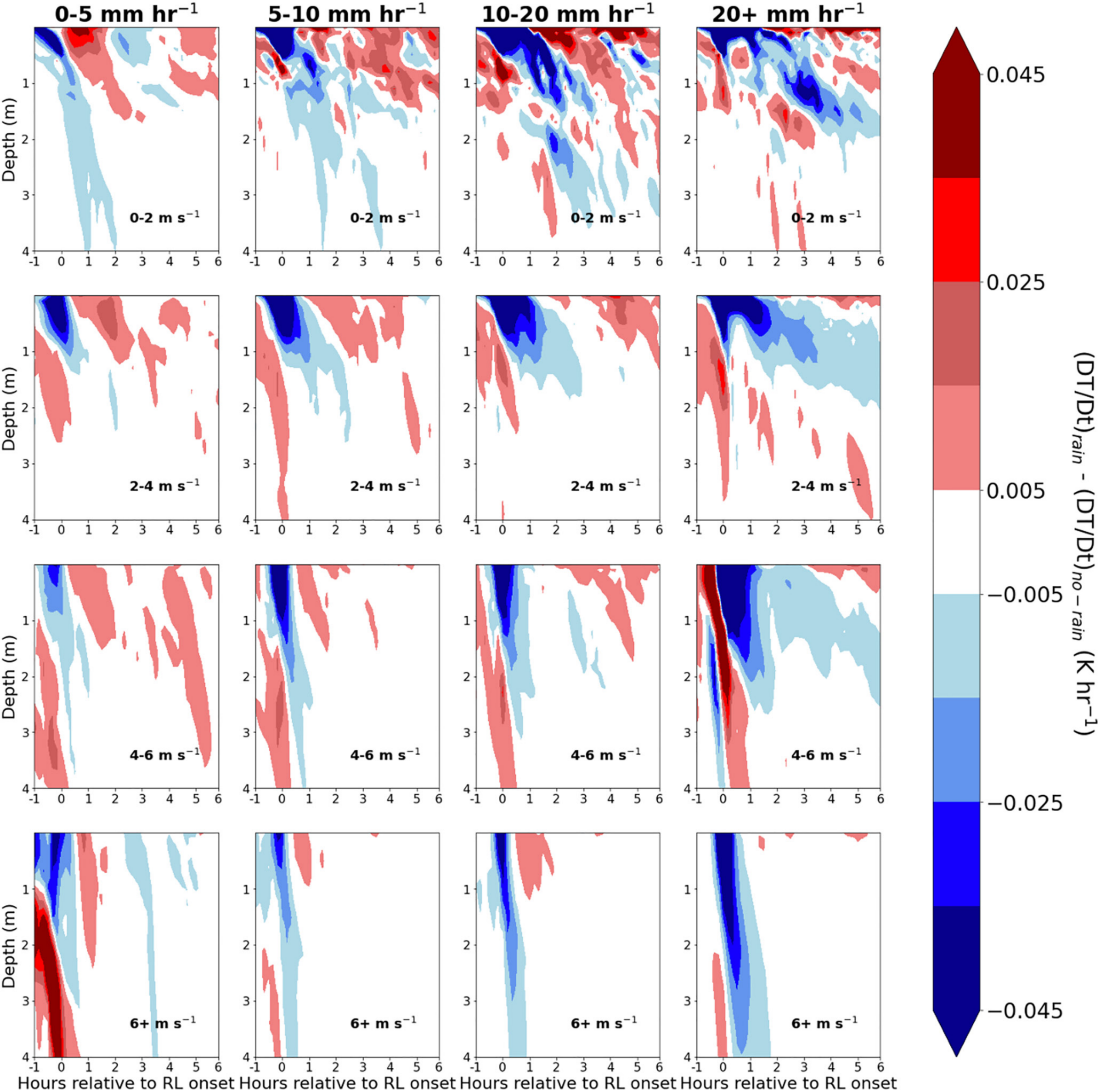
Difference in temperature tendency between simulation with and without precipitation forcing from −1 hr to +6 hr relative to rain layer (RL) onset.

The idea that RL salinity stratification concentrates surface cooling within the RL is supported by differences in total temperature tendency between the RAIN and NO‐RAIN experiments, as shown in Figure 15. Because the temperature tendency from solar heating (the third term in Equation 6) is identical in RAIN and NO‐RAIN, any change in temperature tendency between the rain and no‐rain simulations is the result of a change in the vertical transport term. For all wind speeds and *R*, cooling by vertical transport mixing is reduced following RL onset in RAIN when compared to NO‐RAIN (i.e., red patches following RL onset). This occurs despite the near‐surface unstable temperature stratification that exists within RLs (Figure 8).

### 5.2 RL Feedbacks via Spatial SST Gradients

While the difference in heat fluxes between RAIN and NO‐RAIN is small, Figures 14 and 15 demonstrate that the role of precipitation on SST, and hence SST spatial gradients on the scale of RLs, may be large. Previous studies (Back & Bretherton, 2009a; de Szoeke & Maloney, 2020; Lambaerts et al., 2020; Li & Carbone, 2012) demonstrate that SST gradients force patterns of mass convergence and divergence within the marine boundary layer (MBL) that can initiate atmospheric convection. Here, we explore the role of precipitation in the creation of SST gradients.

Li and Carbone (2012) showed that for the West Pacific warm pool, assuming hydrostatic balance and given the Boussinesq approximation, the time derivative of surface wind convergence is proportional to the Laplacian of the SST field as given by the equation:

(8) $$-{\left(u_{x}+v_{y}\right)}_{t}=w_{z,t}=\frac{p_{xx}^{\prime }+p_{yy}^{\prime }}{{\bar{\rho }}_{b}}=-\frac{gH}{{\bar{T}}_{b}}\left(T_{xx}^{\prime }+T_{yy}^{\prime }\right)$$

where $-{\left(u_{x}+v_{y}\right)}_{t}=w_{z,t}$ is the time derivative of surface wind convergence, *p*′ is the pressure perturbation within a thin layer, *ρ* is air density, *T*′ is the temperature perturbation within the MBL, *H* is the MBL height, and the subscript *b* denotes an environmental mean. SST anomalies influence MBL mass convergence and divergence through the right‐most term in Equation 7, which is the spatial second derivative, or the Laplacian, of *T*′. Because *T*′ is partly set by SST (Back & Bretherton, 2009a), SST gradient contributions to low‐level mass convergence are assessed with the SST Laplacian (∇^2^SST). In their analysis of four years of satellite observations of SST and rainfall, Li and Carbone found that approximately 75% of rainfall events over the West Pacific warm pool were spatially and temporally coincident with local surface convergence maxima, as estimated from the SST Laplacian. Furthermore, the onset of rainfall was more than twice as likely to be observed over −∇^2^SST patches (corresponding to convergence) than over +∇^2^SST patches (corresponding to divergence).

**Figure 16:**
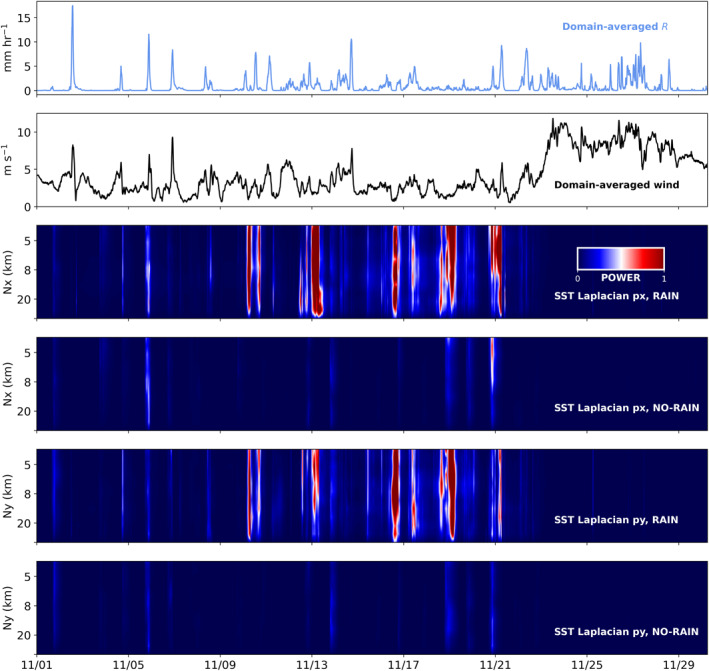
Time series over the one‐month simulation of (top to bottom): domain‐averaged rain rate, domain‐averaged wind speed, domain‐averaged zonal spectral density of sea surface temperature (SST) Laplacian for RAIN, domain‐averaged zonal spectral density of SST Laplacian for NO‐RAIN, domain‐averaged meridional spectral density of SST Laplacian for RAIN, and domain‐averaged meridional spectral density of SST Laplacian for NO‐RAIN. Note: bottom four rows all use the same color bar scale.

To explore the role of RLs in generating SST gradients, we compute the SST Laplacian for the GOTM RAIN and NO‐RAIN simulations. For our analysis, ∇^2^SST is computed at every grid cell using adjacent cells in the model grid. Following Li and Carbone (2012), the SST Laplacian is reported in units of °C per 4 km^2^ to convey the spatial scale of the gradients. Results are presented only for GOTM columns farther than two grid points from the domain boundary to avoid edge effects. Figure 16 displays the temporal evolution of domain‐averaged zonal and meridional spectral density of SST Laplacian for RAIN and NO‐RAIN. In the RAIN experiment, ∇^2^SST has a higher frequency of extreme values than ∇^2^SST in the NO‐RAIN experiment, particularly during periods of increased precipitation and reduced winds (Figure 16). Using the median of the absolute values of ∇^2^SST as an estimate for the width parameter, we find a width parameter of 0.037 for the ∇^2^SST distribution in RAIN, which is nearly double the width parameter of 0.019 in NO‐RAIN. This difference indicates that RLs, through their prolonged reduction of SST compared to adjacent RL‐free columns, are capable of generating sharp SST gradients.

## 6 Discussion

**Figure 17:**
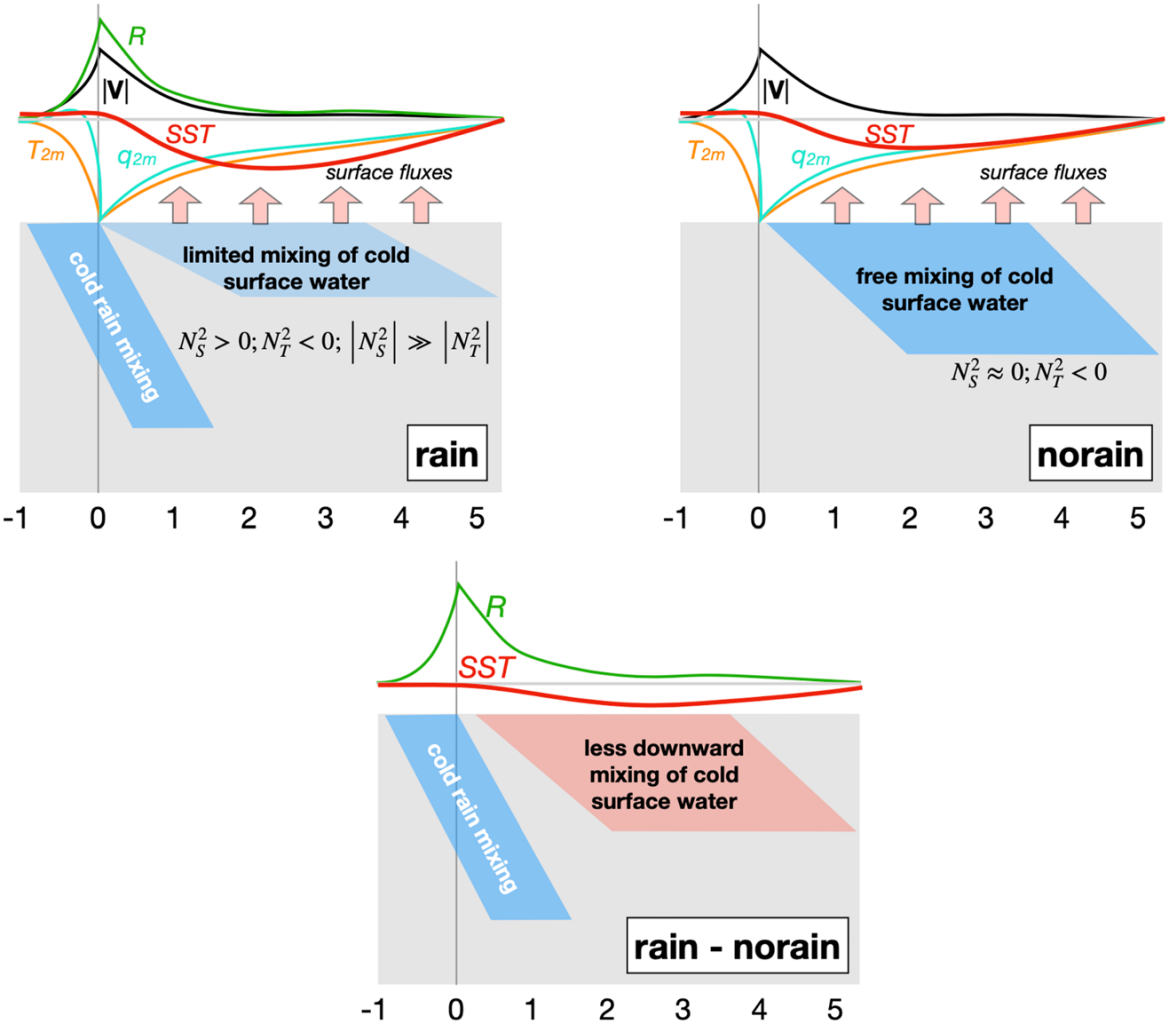
Schematic illustration of atmospheric forcing and ocean response in RAIN (upper left), NO‐RAIN (upper right), and the difference between the two simulations (bottom) for the upper few meters of the ocean and the period one hour prior and five hours following rain layer (RL) onset. Note in the RAIN‐NO‐RAIN panel, the only difference in atmospheric forcing is rainfall, which results in a cold rain input in the upper ocean around RL formation. After the initial cold rain input, the statically stable column in RAIN results in less vertical transport of heat and less subsurface ocean cooling than in NO‐RAIN, confining the coldest water to the surface. Ocean‐to‐atmosphere surface flux differences between RAIN and NO‐RAIN are less than 2% and are omitted from the bottom panel for clarity.

In this section, we synthesize results of the RAIN and NO‐RAIN simulations, and offer some considerations for RL observation and the interpretation of our results. A conceptual aid in the form of a schematic illustration of atmospheric forcing and upper ocean response in RAIN and NO‐RAIN, as well as the differences between the two simulations, can be seen in Figure 17.

For all RLs identified in RAIN, the reduced air temperature and humidity, as well as the increased wind speed and ocean‐to‐atmosphere surface turbulent fluxes following RL onset (Figure 13) are consistent with changes induced by convectively generated cold pools (Feng et al., 2015; Yokoi et al., 2014; Zuidema et al., 2017). The cold rain falling onto the ocean surface and the enhanced surface fluxes contribute to a sustained decrease in SST following RL onset. However, the cold SST signatures are evident even after surface flux perturbations have been restored to their pre‐RL background states, suggesting a role for salinity stratification in regulating SST in RLs.

The NO‐RAIN simulation, which blocks rain from falling onto the ocean surface but otherwise forces the GOTM array with identical surface meteorology as in the RAIN simulation, confirms that salinity stratification by rainfall reduces the SST of RL‐capped columns (Figure 14) by confining ocean water cooling to the near‐surface layer (Figures 8 and 12). Furthermore, when rain falls onto a stably stratified upper ocean, such as onto a DWL, salinity stratification is amplified (Figure 10) and any heat previously accumulated in the temperature‐stratified layer is effectively “hidden” from the atmosphere (Pei et al., 2018; Wijesekera et al., 1999) until the arrival of sufficiently strong winds capable of destroying the RL and mixing the cold surface waters with the warmer subsurface waters (Moum et al., 2014; Thompson et al., 2019).

RLs reduce SST locally, creating a network of SST gradients and boundary layer convergence/divergence patterns that can initiate atmospheric convection. Our study demonstrates that RLs, through their intensification and prolongation of cold SST anomalies, sharpen regional SST gradients and increase the potential for SST gradient‐driven surface convergence to influence atmospheric convection. Thus, RLs may affect the atmosphere in several ways: (a) they prolong locally reduced SST signatures, (b) they shield previously warmed ocean waters from the air‐sea interface, thereby reducing ocean‐to‐atmosphere surface fluxes, and (c) they sharpen regional SST gradients beyond that which can be achieved solely by surface fluxes (Figure 16). It is important to note that the SST gradients and overall domain size in our simulations are much smaller than those in previous studies that connect SST gradients to initiation of atmospheric convection (Li & Carbone, 2012; Skyllingstad et al., 2019). We recommend further studies investigating RL feedback to the atmosphere using ocean‐atmosphere coupled simulations over a larger domain.

Thompson et al. (2019) noted that even rain rates as low as *R* = 5 mm hr^−1^ are capable of forming RLs, and our results (Section 4.1) are consistent with this finding. At low wind speeds, weak *R* cases feature a persistent stable salinity stratification confined to the upper 1 m of the ocean (Figure 9), suggesting that even weakly forced RLs forming under these conditions can last for several hours. Observation of RLs under this forcing regime proves tricky, as stratification is confined to the upper 1–2 m of the ocean and requires high‐resolution near‐surface measurements to capture changes to the water column. The persistence of a stable salinity stratification in weak *R*, low wind speed cases stresses the importance of towed profilers for ship‐based observations that can sample the upper 2–3 m of the ocean outside the ship wake (Drushka et al., 2019) and, thus, capture changes to temperature and salinity under these conditions.

It is important to note that the 1‐dimensional model framework implemented in this study presents a simplified view of ocean dynamics, neglecting the effects of horizontal processes. Lateral advection and propagation of salinity and temperature anomalies associated with RLs distribute SST and SSS anomalies over a greater area and smooths spatial gradients of these variables (Moulin et al., 2021). As such, the extrema of SST gradients and the Laplacian of the SST field in Section 4.4 are likely an overestimate. The absence of lateral advection and the small model domain may also underestimate RL sizes (Figure 10). Larger RLs are hypothesized to occur using time‐space conversion estimates from DYNAMO (Thompson et al., 2019) and almost certainly occur based on the spatial extent of tropical mesoscale convective systems (Houze, 2004). We also note that some studies indicate that GOTM may overestimate SST reduction following precipitation (Pei et al., 2018), although results from our model verification (Section 3) show that GOTM well reproduces observed SST under a variety of atmospheric conditions.

A challenge in using atmospheric model output as forcing data to compile RL statistics arises when assessing RL behavior many hours after formation. Idealized experiments allow for assessment of RL characteristics from single impulse rain rates (Drushka et al., 2016) or an idealized evolution of rain and wind‐based on observations (Iyer & Drushka, 2021a). Forcing GOTM with WRF output provides complex and realistic atmospheric forcing conditions which aids understanding of RL duration, frequency, intensity, and size. Furthermore, the large number of RL‐capped columns sampled over our month‐long simulation allows for composite analysis of RL characteristics as a function of *R* and wind speed surrounding RL onset. However, as surface forcing conditions are constantly changing, it is difficult to account for further freshening events, changes to solar radiation input, high‐frequency wind speed variability, and changes to 2 m specific humidity and temperature, all of which influence RL characteristics minutes to hours after RL formation. Because of these complications, we limit our composite analysis of RL intensity to six hours after RL formation.

Further studies are needed to understand the mesoscale characteristics of RLs globally and over extended periods of time. RL characteristics are determined by rain rate, wind speed, and background ocean stratification, and thus should have a unique presentation in different locations since these factors vary regionally and throughout time. Additionally, as background ocean stratification impacts RL intensity and duration, upper ocean state must be accounted for in RL climatology. Field campaigns that collect collocated, frequently sampled ocean‐atmosphere observations, with fine vertical resolution in the upper ocean, are essential to improving our understanding of RL behavior and impact under different conditions.

## 7 Conclusions and Summary

This study demonstrated that a 1D ocean model (GOTM) effectively replicates observed upper ocean temperature, salinity, and stability profiles in the equatorial Indian Ocean through a comparison with detailed observations of the combined ocean‐atmosphere boundary layers from the DYNAMO field campaign. This result forms the basis for a detailed study on RL statistics from a 2D array of GOTM simulations forced by realistic atmospheric fields from the WRF atmospheric model.

The mean and median RL duration were found to be 4.5 and 1 hr, with a long tail to well over a day, mainly modulated by wind speed. RLs occur very infrequently for wind speeds over 8 m s^−1^, consistent with the findings of Thompson et al. (2019). The RL equivalent diameter is quite uniformly distributed, with larger diameters related to slightly weaker winds on average. RLs reduce mixing due to their stable salinity stratification, which is modulated by the background stratification. RLs often feature unstable temperature stratification due to the low temperature of the initial rain impulse, and further surface cooling by enhanced surface fluxes driven by cold pool atmospheric temperature and humidity anomalies.

RL influence on the air‐sea interactions was studied with a second 2D ocean simulation in which the rain from the atmospheric model was not allowed to fall on the ocean, so no RL formed, but all other atmospheric forcing fields were unchanged. Comparison between the RAIN and NO‐RAIN simulations revealed that the presence of an RL leads to a reduction of SST that persists on time scales longer than the associated rain event. Approximately 1/3 of the SST reduction within RLs can be attributed to rain falling on the ocean surface, and thus, the RL itself, while 2/3 of the SST reduction can be attributed to other atmospheric fields (i.e., wind speed, *q*_air_, *T*_air_, and downward solar radiation). Analysis of SST response in RAIN and NO‐RAIN highlights that RL influence on SST extends well beyond the lifetime of the source rain event. Salinity stratification in the RAIN simulation, and within RLs themselves, inhibits vertical transport of surface cooling to the deeper ocean, yielding SSTs approximately 0.1°C colder than in the NO‐RAIN simulation.

To infer the feedback of the RLs to atmospheric convection, we studied the SST Laplacian, which is directly related to horizonal divergence in the atmosphere boundary layer. Evaluation of the distribution of SST Laplacian for RAIN and NO‐RAIN revealed that the presence of RLs enhances the SST gradients considerably, with the median of the absolute value of the SST Laplacian increased by a factor 2. This result emphasizes the importance of coupled simulations investigating RL feedback to surface fluxes and atmospheric convection.

## Data Availability

Chameleon towed vertical profiler (Moum et al., 2014), and meteorological data collected from the R/V Revelle during the DYNAMO field campaign are available at the Earth Observing Laboratory: https://data.eol.ucar.edu/. Model data was obtained by running the GOTM (Burchard et al., 1999). The model software is publicly available at https://gotm.net/. WRF model output was provided by Samson Hagos from a previous WRF experiment (Hagos et al., 2013). WRF model software is publicly available at https://www2.mmm.ucar.edu/wrf/.
